# Genes optimized by evolution for accurate and fast translation encode in Archaea and Bacteria a broad and characteristic spectrum of protein functions

**DOI:** 10.1186/1471-2164-11-617

**Published:** 2010-11-04

**Authors:** Conrad von Mandach, Rainer Merkl

**Affiliations:** 1 Faculty of Mathematics and Computer Science, University of Hagen, D-58084 Hagen, Germany; 2 Institute of Biophysics and Physical Biochemistry, University of Regensburg, D-93040 Regensburg, Germany

## Abstract

**Background:**

In many microbial genomes, a strong preference for a small number of codons can be observed in genes whose products are needed by the cell in large quantities. This codon usage bias (CUB) improves translational accuracy and speed and is one of several factors optimizing cell growth. Whereas CUB and the overrepresentation of individual proteins have been studied in detail, it is still unclear which high-level metabolic categories are subject to translational optimization in different habitats.

**Results:**

In a systematic study of 388 microbial species, we have identified for each genome a specific subset of genes characterized by a marked CUB, which we named the effectome. As expected, gene products related to protein synthesis are abundant in both archaeal and bacterial effectomes. In addition, enzymes contributing to energy production and gene products involved in protein folding and stabilization are overrepresented. The comparison of genomes from eleven habitats shows that the environment has only a minor effect on the composition of the effectomes. As a paradigmatic example, we detailed the effectome content of 37 bacterial genomes that are most likely exposed to strongest selective pressure towards translational optimization. These effectomes accommodate a broad range of protein functions like enzymes related to glycolysis/gluconeogenesis and the TCA cycle, ATP synthases, aminoacyl-tRNA synthetases, chaperones, proteases that degrade misfolded proteins, protectants against oxidative damage, as well as cold shock and outer membrane proteins.

**Conclusions:**

We made clear that effectomes consist of specific subsets of the proteome being involved in several cellular functions. As expected, some functions are related to cell growth and affect speed and quality of protein synthesis. Additionally, the effectomes contain enzymes of central metabolic pathways and cellular functions sustaining microbial life under stress situations. These findings indicate that cell growth is an important but not the only factor modulating translational accuracy and speed by means of CUB.

## Background

The composition of genes coding for ribosomal proteins and translation elongation factors is highly biased in many genomes [1]. This codon usage bias (CUB) is due to a preference for a species-specific set of codons, which are named major codons. Their particular choice depends on the genomic GC-content and can be explained by amino acid specific rules [2]. Beginning with pioneering work in the 1980s, it has been demonstrated convincingly that major codons are more accurately and more efficiently recognized by the most abundant tRNA species [3-10]. These findings support the hypothesis that major codons are used preferentially in genes coding for proteins required by the cell in large quantities (see [10] and references therein). A further analysis of microbial genomes made clear that CUB is one of several factors to optimize cell growth: Species exposed to selection for rapid growth possess more rRNA operons, more tRNA genes and use major codons more frequently [11,12]. Additionally it turned out that CUB is the best determinant of minimum generation time [13].

Based on different measures of CUB, the occurrence and function of translationally optimized gene products has been studied (see e.g. [14,15] and references therein) and compiled e.g. for *Escherichia coli *[16], *Frankia *[17], or Yeast [18]; however, most reports lack a statistical assessment. Broad multi-species analyses of 27 [19] and 461 microbial genomes [20] aimed at identifying preferred functional categories among codon-optimized genes.

In the following, we report a phylogenetic- and habitat-specific analysis of a particular set of 388 microbial genomes. We found that gene products being optimized for translational efficiency in the course of evolution contribute to protein synthesis, energy production, and protein folding. Compared to Bacteria, translational efficiency is less pronounced in Archaea and restrained to a smaller number of gene functions. In most cases, the function of translationally optimized gene products is only marginally affected by the habitat.

## Results and Discussion

### The GCB-approach constitutes a quantitative measure of translational efficiency for a broad range of genomes

A number of measures for CUB have been used to predict translationally efficient genes in microbial genomes (e.g. [1,21-28]). Generally, H_1_-methods [21] are more suitable to determine the bias associated with translational efficiency than other approaches [29]. A recent comparison of several measures has shown that two H_1_-methods, namely the MELP algorithm [30] and the GCB-approach [31], have the most consistent behaviour for predicting the expression level of individual genes [30]. The GCB-approach, which we utilize in the following, is based on CB-scores determined species-specifically for each codon; see Methods. We first computed these scores for the 912 microbial entries of the NCBI RefSeq database which consists of a curated and non-redundant collection of reference genomes [32]. We have implemented a web-server (accessible *via *http://www-bioinf.uni-regensburg.de) that calculates for a gene sequence the GCB-score for a wide variety of microbial species. GCB-scores take positive and negative values; the more positive a score is, the higher is the fraction of major codons in the considered gene.

**Figure 1:**
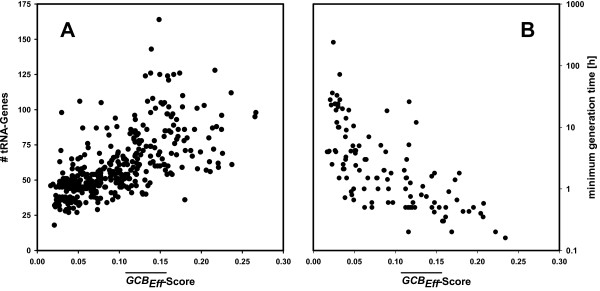
**A plot of $\bar{GCB_{Eff}}$-scores versus the number of tRNA genes and the minimum generation time for microbial species**. Panel A: For 388 microbial species constituting the set *MG_CUB *the number of tRNA genes and the $\bar{GCB_{Eff}}$-score was determined as described and plotted. A Spearman rank correlation confirms the statistically significant correlation of these two values (*r*_*s *_= 0.71, *p *< 0.001). Panel B: The minimum generation time for 113 species of *MG_CUB *(as listed in [13]) was plotted versus the $\bar{GCB_{Eff}}$-score. A Spearman rank correlation confirms the statistically significant correlation of these two values (*r*_*s *_= -0.75, *p *< 0.001).

The numbers of rRNA genes, of tRNA genes, and the genome-wide strength of CUB are highly correlated [11,13]. In order to confirm that GCB-values quantify strength of CUB on the genome level as well, we correlated mean values and the number of tRNA genes in analogy to [11]. The $\bar{GCB_{Eff}}$-value was determined for each genome (see Methods) and taken as a measure for the species-specific strength of CUB. To minimize the risk of false positive classification when identifying translationally optimized genes, we selected those 388 genomes showing a marked CUB (see Additional file 1, Table S1 for a listing and Methods for the selection procedure). A plot of these 388 values *versus *the species-specific number of tRNA genes is shown in Figure 1A. A Spearman rank correlation confirmed for the $\bar{GCB_{Eff}}$-values and the number of tRNA genes a statistically significant correspondence (*r*_*s *_= 0.71, *p *< 0.001), which is stronger than the one deduced from *S *values [11], an alternative measure of CUB. For 113 of these 388 species the minimum generation time is listed in [13]. A plot of these numbers *versus *$\bar{GCB_{Eff}}$-values is shown in Figure 1B. Again, a Spearman rank correlation confirmed a statistically significant correspondence (*r*_*s *_= -0.75, *p *< 0.001), which is stronger than the one published elsewhere [13]. Most likely, the stronger correlation is in both cases due to our focusing on genomes showing a marked CUB.

We conclude from these findings and previous results [30,31] that the GCB-approach allows us to quantify strength of CUB in a consistent manner for a broad range of genes and genomes and to identify translationally optimized genes. Note that we use the term "translationally optimized" for genes showing a marked CUB. As we did not correlate CUB values and mRNA concentrations for a larger set of species, the term optimization as used here is not necessarily related to the expression level of a gene.

In the following, we name the subset of an individual proteome constituted by genes with a GCB-value ≥ 0.0 the *effectome *and the above-introduced set of 388 microbial genomes showing a pronounced codon usage bias *MG_CUB*. Subsets selected for a specific habitat are named *MG_CUB*(*subset*); e.g., *MG_CUB*(Bacteria_TH) comprises the genomes of 13 thermophilic Bacteria belonging to *MG_CUB*.

In a previous study [19] M. Carbone has aimed at characterizing the set of genes deemed to be essential for any given bacterial species. In this context, the set of species-specific genes possessing a marked CUB has been named *functional genomic core*. Although the approach of identifying translationally optimized genes is similar to ours, we did not utilize this term for two reasons: 1) The concept of a *genomic core *has been coined to address the set of intrinsically conserved genes of a phylogenetic group like Archaea (see e.g. [33] and references therein). Thus, the above term might be misinterpreted. 2) Irrespective of the strength of CUB within an individual genome, those 200 genes showing strongest CUB have been analyzed in [19]. In contrast, a species-specific effectome consists of a gene set whose size and composition is exclusively determined by CUB and a well-defined cut off.

Figure 1 shows for Bacteria that the strength of CUB varies markedly among species. A comparison of $\bar{GCB_{Eff}}$-values makes clear that taxonomical position and lifestyle affect the bias: The $\bar{GCB_{Eff}}$-values of mesophilic and thermo-/hyperthermophilic Archaea are similar; the means are 0.070 and 0.074, respectively. In contrast, CUB of psychrophilic/mesophilic Bacteria is higher than that of thermophilic species. The means are 0.10 and 0.06, respectively, and a Mann-Whitney rank sum test signaled the statistically significant difference of the two $\bar{GCB_{Eff}}$-distributions (*p *= 0.003). These findings indicate that CUB is less pronounced in Archaea and strongest in mesophilic and psychrophilic Bacteria.

As pointed out in [13], these differences are most likely due to the dependence of enzyme activity on temperature and might explain CUB in Bacteria. At higher temperature, diffusion increases, viscosity and activation energy decreases, which both facilitate rapid reactions. Therefore, selective strength on CUB is presumably weaker for thermophilic species. In analogy, stronger CUB might be necessary for psychrophilic species to reach a tolerable growth rate.

Species for which speed and efficiency of growth and replication were strong selective forces during evolution are characterized by a high number of tRNA genes [11]. As we expected the widest range of protein functions in the related effectomes, we selected for further analysis those 37 bacterial genomes possessing more than 90 tRNA genes. The composition of the respective subset *MG_CUB*(Bacteria_HITR) is listed in Additional file 1, Table S2. The mean $\bar{GCB_{Eff}}$-value of this set is 0.15 and indicates a strong selective pressure. Concordantly, the mean of minimum generation times for those 14 species of *MG_CUB*(Bacteria_HITR) listed in [13] is 48 min, which is significantly lower than the mean (more than 8 hours) deduced from the whole list.

Depending on the methods used to assess CUB, different fractions of CUB genes have been identified. It has been reported that CUB can be detected in ~28% [34], ~50% [35], ~70% [11], or ~100% [20] of microbial genomes. In the light of these findings, our choice of ~42% of the genomes was a more conservative approach. Here we decided in agreement with [11] and suggest that the lifestyle of a microbe determines the strength of CUB. For species which we did not consider due to a small $\bar{GCB_{Eff}}$-value, we assumed the relative unimportance of exponential growth.

### The effectomes encode a broad and specific range of gene functions

Each analysis of a single proteome reveals a small number of translationally optimized gene products. However, to identify general trends that can be subjected to statistical analyses, one has to explore several genomes and to link the contribution of individual gene products to a more general description of cellular functions. To achieve a multi-level categorization of gene products, we utilized Gene Ontology terms [36] in combination with the classification system of FunCat [37].

Gene Ontology (GO) terms allow the description of gene products by means of a strict vocabulary organized in a hierarchical way. However, assessing the most granular GO-terms used to annotate genes is inappropriate for our purposes: E.g. in *E. coli*, the GO-term "DNA binding" (GO:0004803) is an attribute of transposases, the DnaK suppressor protein, subunits of the DNA polymerase III, elements of prophages, transcription activators, and helicases. Therefore, it is difficult to interpret the overrepresentation of this term in a biologically meaningful way. An overrepresentation of the GO-term "RNA binding" in the effectomes is most probably related to the abundance of ribosomal proteins. These examples demonstrate that higher-level descriptions of gene functions have to be exploited to deduce biologically meaningful results. As an alternative to the analysis of a GO slim (a set of higher level GO-terms) we decided to utilize FunCat categories. FunCat [37] is a functional annotation scheme for the systematic classification of proteins from whole genomes. Utilizing FunCat has an important advantage over GO-terms: As the number of categories needed to classify effectomes is low, we could compare the full composition of the effectomes and the whole genomes by means of robust statistical tests.

**Figure 2:**
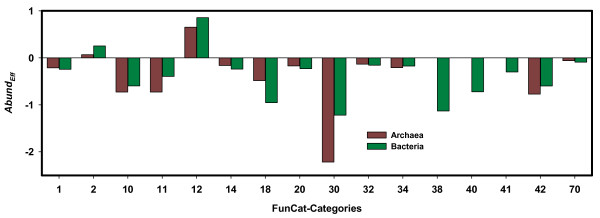
**Abundance of metabolic functions in archaeal and bacterial effectomes**. For all FunCat categories of level 1, the *Abund*_*Eff*_-values were deduced from the datasets *MG_CUB*(Archaea) and *MG_CUB*(Bacteria). Positive scores indicate categories overrepresented in the effectomes. Underrepresented categories have negative values. The categories are numbered according to the FunCat scheme: "metabolism" (1), "energy" (2), "cell cycle and DNA processing" (10), "transcription" (11), "protein synthesis" (12), "protein fate (folding, modification, destination) " (14), "regulation of metabolism and protein function" (18), "cellular transport, transport facilitation and transport routes" (20), "cellular communication/signal transduction mechanism" (30), "cell rescue, defense and virulence" (32), "interaction with the environment" (34), "transposable elements, viral and plasmid proteins" (38), "cell fate" (40), "development (systemic) " (41), "biogenesis of cellular components" (42), "subcellular location" (70). *Abund*_*Eff*_-values were plotted if the number #_*All*_(*Cat*) was at least 100 (compare Table 1).

For an analysis, we deduced for each gene-product GO-terms, mapped them onto high-level FunCat categories and assessed their abundance. Relative frequencies of each category were determined both for a complete dataset *MG_CUB*(*subset*) and the respective effectomes. To quantify the abundance of a category *Cat *within a set of effectomes, we computed the term *Abund*_*Eff*_(*Cat*) which is the log-odds ratio of relative frequencies (see Methods). An *Abund*_*Eff*_(*Cat*) value above zero indicates that *Cat *is overrepresented in the effectomes, a value below zero signals an underrepresentation. To this end, we determined for the set of all archaeal and all bacterial genomes *Abund*_*Eff*_-scores for FunCat categories of level 1, which is the most abstract level of describing protein functions. Results are plotted in Figure 2 and listed in Table 1. These differences in the composition of the effectomes and the underlying whole-genome datasets are statistically significant as confirmed by a chi-square test (*p *< 0.001).

**Table 1:** Abundance of functional categories (FunCat level 1) in archaeal and bacterial effectomes

		Archaea			Bacteria		
**FunCat Category**	**Description**	**#**_***Eff***_**(*Cat*)**	**#**_***All***_**(*Cat*)**	***Abund***_***Eff***_	**#**_***Eff***_**(*Cat*)**	**#**_***All***_**(*Cat*)**	***Abund***_***Eff***_

1	Metabolism	594	15495	-0.21	15948	461317	-0.24

2	Energy	110	1508	0.07	5129	47263	0.25

10	Cell cycle and DNA processing	30	2557	-0.73	1775	116118	-0.60

11	Transcription	32	2746	-0.73	2587	105731	-0.39

12	Protein synthesis	1006	3576	0.65	29446	67653	0.86

14	Protein fate (folding, modification, destination)	91	2106	-0.16	2502	71643	-0.24

18	Regulation of metabolism and protein function	8	395	-0.48	61	8494	-0.95

20	Cellular transport, transport facilitation and transport routes	160	3798	-0.17	5551	155592	-0.23

30	Cellular communication/signal transduction mechanism	0	931	-2.23	138	37418	-1.22

32	Cell rescue, defense and virulence	27	590	-0.13	1026	24273	-0.16

34	Interaction with the environment	21	548	-0.21	1059	26204	-0.17

38	Transposable elements, viral and plasmid proteins	0	68	-	23	5107	-1.13

40	Cell fate	0	9	-	76	6919	-0.72

41	Development (systemic)	0	2	-	3	385	-0.30

42	Biogenesis of cellular components	13	1235	-0.77	1271	83665	-0.60

70	Subcellular localization	191	3511	-0.06	8601	175358	-0.09

	Sum:	2283	39075		75196	1393140	

For archaeal and bacterial genomes, the number of gene products contributing to FunCat categories was determined. The column labeled #_*All*_(*Cat*) gives the number of genes deduced from the whole dataset. The column labeled #_*Eff*_(*Cat*) gives the number of genes belonging to the respective effectomes. The column *Abund*_*Eff *_lists the ratio log(*f*_*Eff *_(*Cat*)/*f*_*All*_(*Cat*)) for the category, if #_*All*_(*Cat*) was at least 100. In all other cases a trend is given indicated by a "-" for underrepresentation. The line labeled Sum lists the number of genes being analyzed.

The comparison of *Abund*_*Eff*_-values indicates a trend towards the translational optimization of several systemic functions. In the following, the number of FunCat categories is given in brackets after their name. As expected, proteins contributing to "protein synthesis" (12) are a major element of the effectomes. In addition, the category "energy" (2) is overrepresented. These findings show that effectomes are to a great extent composed of proteins being related to cell growth and energy production. However, the underrepresentation of "metabolism" (1) and of "transcription" (11) indicates that there is no general trend to optimize translational efficiency of all functions related to cell growth. The categories "cellular communication/signal transduction mechanism" (30), "transposable elements, viral and plasmid proteins" (38) and "regulation of metabolism and protein function" (18) have lowest *Abund*_*Eff*_-values. Most likely, due to their uncritical cellular concentration, elements of regulatory processes (categories 18 and 30) do not undergo optimization of translational efficiency. The codon usage of transposable elements (category 38) and of alien genes is frequently not optimized for their host [38] which explains most likely their underrepresentation in the effectomes. Alternatively, we utilized COG-categories [39] for high level classification because of their different approach of grouping genes. Results are listed in Additional file 1, Table S3 and confirm the general trends. In summary, the analysis reveals a consistent tendency, which is at the systemic level independent of taxonomical position: Both in Archaea and in Bacteria, translationally optimized genes are involved in protein synthesis; additionally they contribute to various cellular functions as e.g. to energy production.

### The habitat has a minor effect on the composition of the effectomes

**Figure 3:**
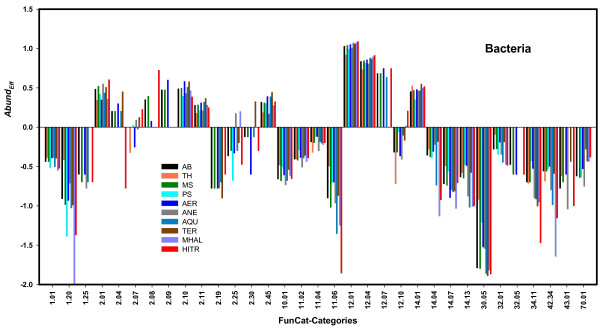
**Habitat specific abundance of metabolic functions in bacterial effectomes**. For FunCat categories of level 2, the *Abund*_*Eff*_-values were deduced for specific bacterial subsets. Scores larger than zero indicate categories overrepresented in the effectomes. Underrepresented categories have negative values. The categories are numbered according to the FunCat scheme: "amino acid metabolism" (1.01), "secondary metabolism" (1.20), "extracellular metabolism" (1.25), "glycolysis and gluconeogenesis" (2.01), "glyoxylate cycle" (2.04), "pentose-phosphate pathway" (2.07), "pyruvate dehydrogenase complex" (2.08), "anaplerotic reactions" (2.09), "tricarboxylic-acid pathway (citrate cycle, Krebs cycle, TCA cycle)" (2.10), "electron transport and membrane-associated energy conservation" (2.11), "metabolism of energy reserves (e.g. glycogen, trehalose)" (2.19), "oxidation of fatty acids" (2.25), "photosynthesis" (2.30), "energy conversion and regeneration" (2.45), "DNA processing " (10.01), "RNA synthesis" (11.02), "RNA processing" (11.04), "RNA modification" (11.06), "ribosome biogenesis" (12.01), "translation" (12.04), "translational control" (12.07), "aminoacyl-tRNA synthetases" (12.10), "protein folding and stabilization" (14.01), "protein targeting, sorting and translocation" (14.04), "protein modification" (14.07), "protein/peptide degradation" (14.13), "transmembrane signal transduction" (30.05), "stress response" (32.01), "disease, virulence and defense" (32.05), "cellular sensing and response to external stimulus" (34.11), "prokaryotic cell envelope structures" (42.34), "fungal/microorganismic cell type differentiation" (43.01), "cell wall" (70.01). *Abund*_*Eff *_-values were plotted if the number #All(*Cat*) was at least 100. Abbreviations of subsets: AB all, TH thermophilic, MS mesophilic, PS psychrophilic, AER aerobic, ANE anaerobic, AQU aquatic, TER terrestrial, MHAL moderately halophilic bacteria, and HITR the subset of Bacteria possessing an extreme number of tRNA genes represented by *MG_CUB*(Bacteria_HITR).

To study the impact of the habitat on the composition of effectomes, we determined *Abund*_*Eff*_-values for the set of all archaeal and all bacterial effectomes, for *MG_CUB*(Bacteria_HITR) and for subsets of hyperthermophilic, thermophilic, mesophilic, psychrophilic, aquatic, terrestrial, host-associated, aerobic, anaerobic, non-halophilic, and moderately halophilic Archaea or Bacteria contributing to *MG_CUB*, if the subset contained at least seven genomes; see Additional file 1, Table S4. For a more detailed analysis of the effectomes and to corroborate the overrepresentation of specific functions not detectable at FunCat level 1, we determined *Abund*_*Eff*_-values for FunCat categories of level 2 and compiled them in Additional file 2. Table 2 lists for 12 habitats categories overrepresented in at least one subset of archaeal or bacterial effectomes. In agreement with the above findings, sub-categories related to "protein synthesis" (12.01, 12.04, 12.07) are overrepresented in archaeal and bacterial effectomes. Additionally, specific functions belonging to "protein folding and stabilization" (14.01) are overrepresented both in bacterial and archaeal effectomes. Compared to Bacteria, archaeal effectomes contain a smaller number of gene products related to energy production. In bacterial effectomes enzymes being parts of "glycolysis and gluconeogenesis" (2.01) and of the "tricarboxylic-acid pathway (citrate cycle, Krebs cycle, TCA cycle)" (2.10) are the dominating elements of energy production. All other protein functions are less overrepresented in bacterial effectomes. As expected, *Abund*_*Eff*_-values of bacterial genomes being most optimized for cell growth [represented by *MG_CUB*(Bacteria_HITR)] are in many cases most extreme (compare Table 2) and deviate in some cases from general tendencies. In summary, a comparison of the *Abund*_*Eff*_-values indicates two general trends: 1) The composition of archaeal effectomes is focused on a smaller number of systemic gene functions. 2) The habitat has only a minor effect on effectome composition. Figure 3 illustrates the latter finding for nine bacterial habitats: In nearly all cases, the strength of over- or underrepresentation is similarly high.

**Table 2:** Functional categories (FunCat level 2) and their abundance in archaeal and bacterial effectomes

FunCat Category	Description	Archaea				Bacteria											
		HT	MS	AQU	ANE	AB	TH	MS	PS	AQU	TER	HOA	AER	ANE	NHAL	MHAL	HITR

2.01	Glycolysis and gluconeogenesis	+	-0.32	-0.01	-0.17	0.49	0.34	0.52	0.42	0.44	0.51	0.46	0.35	0.55	0.56	0.36	0.60

2.04	Glyoxylate cycle	x	x	x	x	0.20	+	0.20	+	0.20	0.45	-0.12	0.30	+	0.34	+	-0.78

2.07	Pentose-phosphate pathway	-	-	-	-	0.00	-0.32	0.00	0.03	-0.03	0.12	-0.12	-0.26	0.09	0.04		0.23

2.08	Pyruvate dehydrogenase complex	x	x	x	x	0.35	-	0.40	+	+	+	0.40	0.08	x	0.60	+	0.73

2.09	Anaplerotic reactions	x	x	x	x	0.48	+	0.48	+	+	+	+	0.60	+	+	+	0.00

2.10	Tricarboxylic-acid pathway (citrate cycle, Krebs cycle, TCA cycle)	x	+	+	x	0.49	+	0.50	0.40	0.51	0.58	0.37	0.58	0.43	0.49	0.46	0.39

2.11	Electron transport and membrane-associated energy conservation	0.10	0.30	0.10	0.25	0.28	0.18	0.28	0.21	0.32	0.37	0.22	0.31	0.21	0.26	0.28	0.25

2.30	Photosynthesis	x	x	x	x	-0.12	-	-0.12	x	-0.12	0.33	-0.48	-0.60	+	-0.48	x	-0.30

2.45	Energy conversion and regeneration	+	0.29	+	+	0.32	0.19	0.31	0.30	0.39	0.44	0.23	0.39	0.17	0.29	0.27	0.33

12.01	Ribosome biogenesis	0.79	0.85	0.79	0.82	1.03	0.92	1.04	1.00	1.07	1.06	0.94	1.05	1.00	1.07	1.08	1.10

12.04	Translation	0.63	0.66	0.62	0.65	0.84	0.73	0.85	0.82	0.88	0.87	0.75	0.86	0.81	0.87	0.90	0.91

12.07	Translational control	+	+	+	+	0.68	+	0.68	+	0.64	+	0.65	0.75	+	0.74	+	0.75

12.10	Aminoacyl-tRNA synthetases	-	-	-	-	-0.32	-	-0.32	-	-	-	-	-	-	-	-	0.21

14.01	Protein folding and stabilization	+	0.46	+	+	0.45	0.53	0.47	0.35	0.46	0.55	0.36	0.48	0.46	0.46	0.50	0.52

32.07	Detoxification	+	-	+	+	0.09	0.20	0.09	0.10	0.11	0.20	-0.01	0.09	0.00	0.12	0.15	0.26

34.01	Homeostasis	+	-	+	+	0.09	-0.02	0.11	0.02	0.13	0.12	0.03	0.07	0.10	0.07	0.02	0.22

34.05	Cell motility	-	+	0.08	0.01	-0.04	-0.08	-0.02	-0.22	-0.25	0.16	0.17	0.12	0.06	-0.09	-0.38	-0.50

42.33	Pilus/fimbria	x	x	x	x	0.00	0	0.00	+	+	0	-0.40	0.18	-	0.00	+	0.00

70.03	Cytoplasm	x	x	x	x	0.02	-0.07	0.03	0.03	0.04	0.08	-0.02	0.05	-0.01	0.04	0.05	0.10

70.27	Extracellular/secretion proteins	+	-	+	+	0.34	+	0.36	+	0.18	0.22	0.39	0.28	0.40	0.44	+	0.26

70.34	Prokaryotic cell envelope component	x	x	x	x	0.05	x	0.07	0.02	0.01	0.04	0.09	-0.04	-0.14	0.17	0.07	0.12

For archaeal and bacterial genomes, the number of gene products contributing to FunCat categories of level 2 was determined (see Methods). The columns lists the ratio log(*f*_*Eff *_(*Cat*)/*f*_*All *_(*Cat*)) for the categories, if #_*All*_(*Cat*) was at least 100 (compare Table 1). In all other cases a trend is given indicated by a "+" for overrepresentation, a "-" for underrepresentation and a 0 for a score value of 0.0. An "x" indicates categories not occurring in the respective dataset. Abbreviations of subsets: AB all Bacteria, HT hyperthermophilic, TH thermophilic, MS mesophilic, PS psychrophilic, AQU aquatic, TER terrestrial, HOA host-associated, AER aerobic, ANE anaerobic, NHAL non halophilic, MHAL moderately halophilic species, and HITR the subset of bacterial species possessing an extreme number of tRNA genes, *i.e. MG_CUB*(Bacteria_HITR).

### A paradigmatic case: The effectome composition of bacterial genomes being strongly optimized for cell growth

In order to analyze effectome composition on the level of individual gene products, we used the eggNOG database [40], which consists of functionally annotated clusters of orthologous genes (COGs) [39]. Additionally, we mapped enzymes onto reference pathways of the KEGG database [41]. To study a prominent example, we analyzed the effectomes of those Bacteria which show strongest signals of translational optimization [the set *MG_CUB*(Bacteria_HITR)]. The composition of these effectomes is compiled in Additional file 3; respective identifiers for the eggNOG and KEGG database are listed in Additional file 4. Some examples that substantiate the broad range of gene functions contributing to these effectomes are given in the following list, which is sorted according to FunCat categories and annotated according to eggNOG.

#### Glycolysis and gluconeogenesis (2.01)

Enolases, which are essential for the degradation of carbohydrates *via *glycolysis; other enzymes of central pathways like glyceraldehyde-3-phosphate dehydrogenase/erythrose-4-phosphate dehydrogenase; fructose/tagatose bisphosphate aldolase; the pyruvate/2-oxoglutarate dehydrogenase complex; triosephosphate isomerase; 3-phosphoglycerate kinase.

#### Tricarboxylic-acid pathway (citrate cycle, Krebs cycle, TCA cycle) (2.10)

The succinyl-CoA synthetase.

#### Electron transport and membrane-associated energy conservation (2.11)

Elements of the F0F1-type ATP synthase.

#### Ribosome biogenesis (12.01)

All ribosomal proteins of both subunits.

#### Translation (12.04)

Translation initiation factors 1, 2, 3; translation elongation factors Tu, Ts, P; the ribosome recycling factor; aminoacyl-tRNA synthetases (see 12.1); ribosomal proteins (see 12.01).

#### Translational control (12.07)

Bacterial nucleoid DNA-binding protein.

#### Aminoacyl-tRNA synthetases (12.1)

Synthetases transferring 16 different amino acids occur in the effectomes. The missing tRNA synthetases are related to Gln, His, Cys and Trp.

#### Protein folding and stabilization (14.01)

Several proteins involved in protein folding and stabilization like chaperones; the peptidyl-prolyl cis-trans isomerase (rotamase), which accelerates the folding of proteins; the parvulin-like peptidyl-prolyl isomerase, which plays a major role in protein secretion; the protease subunit of ATP-dependent Clp proteases, which are important for the degradation of misfolded proteins; the cell division GTPase, which is essential for the cell-division process.

#### Detoxification (32.07)

Superoxide dismutase, destroying radicals which are normally produced within the cells and which are toxic to biological systems.

#### Homeostasis (34.01)

The DNA-binding ferritin-like protein, which protects DNA from oxidative damage.

#### Cytoplasm (70.03)

Cold shock proteins, inhibiting DNA replication at both initiation and elongation steps; the pleiotropic transcriptional repressor, which represses the expression of many genes that are induced as cells make the transition from rapid exponential growth to stationary phase; elements of the glycine cleavage system, which catalyzes the degradation of glycine; glycine/serine hydroxymethyltransferase, which supports the interconversion of serine and glycine; nucleoside diphosphate kinase, which is involved in the synthesis of nucleoside triphosphates other than ATP; adenylosuccinate synthase, which belongs to the *de novo *pathway of purine nucleotide biosynthesis; several outer membrane proteins.

The mapping of enzymes belonging to FunCat categories 2.01, 2.10, and 2.11 onto KEGG reference pathways makes clear that all enzymes constituting the core of the glycolysis/gluconeogenesis pathway and the TCA cycle are elements of these effectomes; see Additional file 5, Figure S1 and Additional file 6, Figure S2.

### The analysis of multiple genomes allows a fine grained correlation of CUB and gene functions

Due to the small number of CUB genes being identified in a single genome, former analyses of individual genomes or small sets of related species (see e.g. [14]) could identify only a small set of individual gene functions being translationally optimized. These results have been confirmed by [19,20] and our findings. These three multi-species analyses agree in detecting an overrepresentation of translationally optimized genes in central metabolic functions like in protein synthesis or energy production. However, for other high level functions, some findings presented here and in [19] or [20] differ.

Considering individual genes, many of our results coincide with the outcome of [19], which is based on a smaller set of genomes. This is also true for less pronounced gene functions like the elements of the photosynthesis system of *Synechocystis*, the role of ferredoxin in *Pyrococcus abyssi *and the central enzymes of methane metabolism in *Methanosarcina acetivorans*. In contrast, all proteins involved in acetoclastic methanogenesis [42] do not belong to the effectome of *M. acetivorans*, as their GCB-value is ≤ -0.03. The conclusions drawn on the level of metabolic pathways are contrary in some cases, too. For example, in the effectomes of Archaea and Bacteria elements of the transcription apparatus (FunCat category 11) and of transmembrane signal transduction (FunCat category 30.05) are significantly underrepresented, which is in contrast to the postulated composition of functional genomic cores [19]. Our approach regards a metabolic function as translationally optimized only if more than the expected number of related genes shows a marked CUB. It is a matter of debate whether CUB in a small number of related genes is sufficient to declare a whole metabolic process as translationally optimized.

A recently published study [20] has been based on a machine learning approach for the identification of genes possessing an optimized codon usage (OCU). At mean, the considered genomes have contained 13.2% of OCU genes, in extreme cases, 33% of the genomic content has been OCU. These genes have been utilized to corroborate the enrichment or depletion of metabolic functions which have been characterized by means of GO-terms. In contrast, the effectomes analyzed here, are much smaller: 86% of the effectomes are constituted by at most 5% of the respective genomic content; only four *Borrelia *species possess effectomes containing more than 25% of their genes. Despite these differences in the amount of CUB genes, the outcomes of both studies overlap to a great extent considering high-level metabolic functions. For example, "electron transport and membrane associated energy conservation" (FunCat category 2.11) and the respective GO-term "ATP synthesis coupled proton transport" were reported as overrepresented. The same is true for functions related to protein folding and elements of energy production like the TCA cycle. Both studies identify an underrepresentation of functions related to "DNA repair" and "inorganic ion transport" (see Additional file 1, Table S3). On the other hand, an enrichment of functions related to antibiotic biosynthesis, nitrogen fixation and of iron-sulfur cluster assembly has only been observed among OCU genes.

Most interestingly, both analyses made clear that the habitat has only a little effect on the set of translationally optimized genes. The habitat-specific analyses did not identify an additional translationally optimized high-level metabolic function. However, considering more specific functions, some habitat-specific findings differ. For example, the overrepresentation of aminoacyl-tRNA synthetases was only identified for *MG_CUB*(Bacteria_HITR). Most plausible, these disparities as well as those of enrichment/depletion factors are due to the approach-specific choice of analysed gene sets: Effectomes contain exclusively genes showing a marked CUB found in a small set of genomes whereas OCU genes are larger subsets of genomes and have been recruited from a larger set of species. This might e.g. explain why the overrepresentation of genes related to bacterial chromatin is much lower in the effectomes than among OCU genes. The ratios of enrichment factors are 1.34/6.43 for Fis, 1.27/6.21, for IHF, and 1.64/3.82 for Dps, respectively. On the other hand, the maximal enrichment factor for GO-terms among bacterial OCU genes is 8.3. In bacterial effectomes "ribosomal biogenesis" is overrepresented more than 10-fold and "cellular communication" and "transposable elements, viral and plasmid proteins" are depleted more than 10-fold. These differences suggest as future work a more detailed analysis of translationally optimized genes categorized according to the individual strength of CUB.

### The analysis of effectomes contributes to a more detailed understanding of critical conditions in microbial life

Most of our knowledge about molecular biology and the physiology of microorganisms has been deduced from batch culture, chemostats, and turbidostats. However, this state of balanced growth is completely unnatural for practically all microbes [43]. In many natural habitats nutrients and energy supplies are limited most of the time. This is why microbes exist in a continuous state of starvation and are in addition competing with other microorganisms for survival. It is difficult to simulate such situations in wet-lab experiments.

In contrast, CUB is the result of selection that shapes individual genomes on an evolutionary timescale. Thus, analysing CUB allows the identification of cellular functions requiring the optimization of translational efficiency in the natural environment. This is why the composition of the effectomes indicates critical elements of metabolic functions and identifies proteins whose translational accuracy and speed is crucial in situations occurring frequently in the typical microbial habitat.

Knowing these critical functions is an important value in itself, but this knowledge might also be relevant for the tailoring of productive strains. For example, our analysis of bacterial genomes being strongly optimized for cell growth made clear that aminoacyl-tRNA synthetases are overrepresented in the respective effectomes. If related strains are used for protein production, it is plausible to assess codon usage and the *in vivo *concentration of these enzymes in order to maximize the yield.

A comparison of $\bar{GCB_{Eff}}$-values and the composition of the effectomes highlight a consistent trend: Generally and independent of the strength of CUB, several central functions involved in protein synthesis, energy production, and protein folding are translationally optimized. Additionally, in certain habitats and due to the prevalent selective forces, both the strength of CUB and the palette of translationally optimized gene products increase. This hypothesis is supported by the above mentioned overrepresentation of aminoacyl-tRNA synthetases. The effectomes of *MG_CUB*(Bacteria_HITR) contain tRNA synthetases that load 16 different amino acids. Most plausibly, three synthetases do not occur in the effectomes because they are related to amino acids (Trp, Cys, His), which are rare in microbial proteins. The fourth and last aminoacyl-tRNA-synthetase missing in the effectomes is tRNA(Gln). In several Bacteria, Gln-tRNA^Gln ^is produced by means of a mischarged Glu-tRNA^Gln ^and a Glu-tRNA^Gln ^amidotransferase (consisting of subunits A, B, C) through the transamidation of misacylated Glu-tRNA^Gln ^[44]. Due to the small number of genes, a statistically sound analysis is not possible in this case. However, in the genomes of *Bacillus cereus, Bacillus anthracis*, and *Bacillus thuringiensis*, which lack a glutaminyl-tRNA synthetase, the large subunits A and B of the aspartyl/glutamyl-tRNA(Asn/Gln) amidotransferase have only slightly negative GCB-values (-0.05 and -0.08, respectively). This finding is a further indicator for the fine-tuned composition of microbial effectomes.

In competitive environments nature has found many ways of improving cell growth and response times. A stunning example is the distinctive codirectionality of replication and transcription as e.g. seen in *Clostridium tetani*. 82% of the genes are transcribed in the same direction as DNA replication [45]. Along these lines, our findings highlight a further facet of the complexity of microbial genomes, their composition, and regulation by confirming the importance of translational efficiency for a large number of protein functions.

## Conclusions

### Cell growth is an important but not the only factor modulating translational efficiency

Definitely, the optimization of protein synthesis is the strongest selective factor dominating the composition of effectomes. This statement is confirmed by the finding that aminoacyl-tRNA synthetases loading abundant amino acids have been optimized by evolution for translational accuracy and speed. However, the underrepresentation of protein functions involved in transcription and metabolism makes clear that only a specific subset of functions related to cell growth are subject to translational optimization. Our results show that several selective forces modulate the level of translational efficiency. This hypothesis is confirmed by the overrepresentation in the effectomes of chaperones, which assist protein folding, and of proteases, which degrade misfolded proteins. Minimizing damage due to radicals and oxygen as well as the rapid control of DNA replication and gene expression are additional and crucial tasks supported by translationally optimized gene products.

## Methods

### *MG_CUB*, a non-redundant set of microbial genomes with a marked CUB

We used the microbial genomes section of the Reference Sequence database (RefSeq, version as of Feb. 2009, 912 replicons) [32] to access a non-redundant collection of richly annotated chromosomes. To concentrate on species with a marked CUB that indicates translational efficiency, we selected datasets containing at least five ribosomal genes with a GCB-value ≥ 0.0 and at least one gene with a GCB-value ≥ 0.1. After eliminating entries belonging to the same taxonomical genus, the complete dataset *MG_CUB *contained 388 microbial genomes (see Additional file 1, Table S1). The subset of bacterial genomes *MG_CUB*(Bacteria) subsumes 370 entries with 1 175 058 genes. The subset *MG_CUB*(Archaea) contains 18 genomes and 39 092 genes. Analogously, subsets containing habitat- or taxon-specific groups *HS *were named *MG_CUB*(*taxon*_*HS*); e.g., *MG_CUB*(Bacteria_TH) is the subset of genomes from thermophilic Bacteria; see Additional file 1, Table S4 for details. All subsets analyzed here contain at least seven genomes. The habitat of the microbes was taken from the file ftp://ftp.ncbi.nlm.nih.gov/genomes/Bacteria/lproks_0.txt. Minimum generation times are from [13].

### Determination of GCB-values

The GCB-approach follows the classical and proven concepts of scoring-functions as e.g. utilized for sequence comparison [46,47] or the identification of horizontally transferred genes [48]. It is based on a species-specific set of codon bias (CB) scores. A CB-score is defined as

$$CB_{j}(cdn_{i})=\log(\frac{f_{ref}^{j}(cdn_{i})}{f_{mean}^{j}(cdn_{i})}).$$

Here $f_{{}_{mean}}^{j}(cdn_{i})$ is the mean frequency of codon *cdn*_*i *_in the genome of species *j *and $f_{{}_{ref}}^{j}(cdn_{i})$ is the codon usage in a set of reference genes from genome *j*. As has been shown, ribosomal genes are a valid starting point to determine reference frequencies [30,49]. In order to deduce CB-scores from genes with strongest bias, the GCB-approach initially starts with codon frequencies of ribosomal genes and utilizes a steepest gradient method to iteratively improve the CB-scores similarly to the concept of [27]. Using CB-values, the GCB-score of an individual gene from species *j *is determined as

$$\begin{array}{c} GCB(gene,j)=GCB(cdn_{1},cdn_{2},...,cdn_{n},j)\\ =\frac{1}{n}\sum_{i=1}^{n}CB_{j}(cdn_{i}). \end{array}$$

As a measure for the strength of CUB in a genome *j*, we utilize the mean

$$\bar{GCB_{Eff}(j)}=\frac{1}{m}\sum_{k=1}^{m}GCB(gene_{k},j)$$

deduced from the *m *genes of genome *j *with *GCB*(*gene*_*k,*_*j*) ≥ 0.0, i.e. the species-specific effectome. By limiting the calculation to the content of the effectome, we avoid the likely distortion of the mean value caused by horizontally acquired genes. Due to their origin, most alien genes possess an unrelated codon usage [38]. Thus, a mean GCB-value inferred from the whole genome depends on the origin and the fraction of alien genes, which might render useless this indicator of translational efficiency.

### Mapping genes to FunCat and scoring the abundance of categories

For each gene product, GO-terms [36] were used to relate the product to FunCat categories [37]. The FunCat system is a one to many mapping of individual gene products to functional categories. For that reason, we have not reported on categories like "protein with binding function or cofactor requirement (structural or catalytic)" (16), which are in the case of effectomes dominated by ribosomal proteins. For a taxon- or habitat-specific set of genomes *MG_CUB*(*taxon*_*HS*), the number of gene products contributing to each FunCat category *Cat *was determined both for the whole dataset (#_*All*_(*Cat*)) and those genes belonging to the related effectomes (#_*Eff *_(*Cat*)). A log-odds score *Abund*_*Eff *_was deduced from the resulting frequencies *f*_*All*_(*Cat*) and *f*_*Eff *_(*Cat*) as

$$Abund_{Eff}(Cat)=\log(f_{Eff}(Cat)/f_{All}(Cat)).$$

A log-odds ratio above zero indicates that more than the number of genes expected due to the distribution of categories in the whole dataset occurs in the effectome. *Abund*_*Eff*_-values quantify over- and underrepresentation of categories symmetrically about zero according to log(*a*) = -log(1/*a*). For a two-fold over- and the respective underrepresentation follows: log(2) = 0.30 and log(1/2) = -0.30. In order to avoid outliers caused by a too small number of samples, we only analyzed subsets that contained at least seven genomes.

### Determining the function of individual proteins

The UniProt interface [50] was used to map RefSeq identifiers of individual genes onto UniProtKB accession numbers which were fed into the eggNOG database [40]. Thus, we deduced for a set of genes from different genomes a categorized description of protein function in terms of COG classes [39]. Based on RefSeq identifiers, KO-numbers were determined, mapped onto KEG reference pathways [41], and plotted color-coded.

## Authors' contributions

CvM carried out the computational analysis. RM conceived the study, did the statistical analysis and drafted the manuscript. All authors read and approved the final manuscript.

## Supplementary Material

Additional file 1 **Tables S1 - S4**. Click here for file

Additional file 2 **Excel spreadsheets listing the composition of effectomes for all habitats on FunCat level 2**. Click here for file

Additional file 3 **Excel spreadsheet containing the gene names of the effectomes of extremely optimized Bacteria**. Click here for file

Additional file 4 **Excel spreadsheet containing the COG- and KO-numbers of genes from the effectomes of extremely optimized Bacteria**. Click here for file

Additional file 5 **Figure S1 - Glycolysis/Gluconeogenesis as depicted in the reference pathway of KEGG**. Click here for file

Additional file 6 **Figure S2 - TCA cycle as depicted in the reference pathway of KEGG**. Click here for file

## References

[B1] Sharp PM Li WH The codon adaptation index - a measure of directional synonymous codon usage bias, and its potential applications Nucleic Acids Res 1987 15 3 1281 1295 10.1093/nar/15.3.1281 3547335 PMC340524

[B2] Hershberg R Petrov DA General rules for optimal codon choice PLoS Genet 2009 5 7 e1000556 10.1371/journal.pgen.1000556 19593368 PMC2700274

[B3] Bennetzen JL Hall BD Codon selection in yeast J Biol Chem 1982 257 6 3026 3031 7037777

[B4] Gouy M Gautier C Codon usage in bacteria: correlation with gene expressivity Nucleic Acids Res 1982 10 22 7055 7074 10.1093/nar/10.22.7055 6760125 PMC326988

[B5] Grantham R Gautier C Gouy M Mercier R Pave A Codon catalog usage and the genome hypothesis Nucleic Acids Res 1980 8 1 r49 r62 10.1093/nar/8.1.197-c 6986610 PMC327256

[B6] Sharp PM Li WH An evolutionary perspective on synonymous codon usage in unicellular organisms J Mol Evol 1986 24 1-2 28 38 10.1007/BF02099948 3104616

[B7] Ikemura T Correlation between the abundance of *Escherichia coli *transfer RNAs and the occurrence of the respective codons in its protein genes J Mol Biol 1981 146 1 1 21 10.1016/0022-2836(81)90363-6 6167728

[B8] Curran JF Yarus M Rates of aminoacyl-tRNA selection at 29 sense codons in vivo J Mol Biol 1989 209 1 65 77 10.1016/0022-2836(89)90170-8 2478714

[B9] Pedersen S *Escherichia coli *ribosomes translate in vivo with variable rate EMBO J 1984 3 12 2895 2898 6396082 10.1002/j.1460-2075.1984.tb02227.x PMC557784

[B10] Andersson SG Kurland CG Codon preferences in free-living microorganisms Microbiol Rev 1990 54 2 198 210 2194095 10.1128/mr.54.2.198-210.1990 PMC372768

[B11] Sharp PM Bailes E Grocock RJ Peden JF Sockett RE Variation in the strength of selected codon usage bias among bacteria Nucleic Acids Res 2005 33 4 1141 1153 10.1093/nar/gki242 15728743 PMC549432

[B12] Rocha EP Codon usage bias from tRNA's point of view: redundancy, specialization, and efficient decoding for translation optimization Genome Res 2004 14 11 2279 2286 10.1101/gr.2896904 15479947 PMC525687

[B13] Vieira-Silva S Rocha EP The systemic imprint of growth and its uses in ecological (meta)genomics PLoS Genet 2010 6 1 e1000808 10.1371/journal.pgen.1000808 20090831 PMC2797632

[B14] Karlin S Brocchieri L Campbell A Cyert M Mrazek J Genomic and proteomic comparisons between bacterial and archaeal genomes and related comparisons with the yeast and fly genomes Proc Natl Acad Sci USA 2005 102 20 7309 7314 10.1073/pnas.0502314102 15883367 PMC1129125

[B15] Karlin S Brocchieri L Mrázek J Kaiser D Distinguishing features of d-proteobacterial genomes Proc Natl Acad Sci USA 2006 103 30 11352 11357 10.1073/pnas.0604311103 16844781 PMC1544090

[B16] Roymondal U Das S Sahoo S Predicting gene expression level from relative codon usage bias: an application to *Escherichia coli *genome DNA Res 2009 16 1 13 30 10.1093/dnares/dsn029 19131380 PMC2646356

[B17] Sen A Sur S Bothra AK Benson DR Normand P Tisa LS The implication of life style on codon usage patterns and predicted highly expressed genes for three *Frankia *genomes Antonie Van Leeuwenhoek 2008 93 4 335 346 10.1007/s10482-007-9211-1 18293096

[B18] Das S Roymondal U Sahoo S Analyzing gene expression from relative codon usage bias in Yeast genome: a statistical significance and biological relevance Gene 2009 443 1-2 121 131 10.1016/j.gene.2009.04.022 19410638

[B19] Carbone A Computational prediction of genomic functional cores specific to different microbes J Mol Evol 2006 63 6 733 746 10.1007/s00239-005-0250-9 17103060

[B20] Supek F Skunca N Repar J Vlahovicek K Smuc T Translational selection is ubiquitous in prokaryotes PLoS Genet 2010 6 6 e1001004 10.1371/journal.pgen.1001004 20585573 PMC2891978

[B21] Wright F The 'effective number of codons' used in a gene Gene 1990 87 1 23 29 10.1016/0378-1119(90)90491-9 2110097

[B22] Morton BR Codon use and the rate of divergence of land plant chloroplast genes Mol Biol Evol 1994 11 2 231 238 8170364 10.1093/oxfordjournals.molbev.a040105

[B23] Freire-Picos MA Gonzalez-Siso MI Rodriguez-Belmonte E Rodriguez-Torres AM Ramil E Cerdan ME Codon usage in *Kluyveromyces lactis *and in yeast cytochrome c-encoding genes Gene 1994 139 1 43 49 10.1016/0378-1119(94)90521-5 8112587

[B24] Karlin S Mrázek J Predicted highly expressed genes of diverse prokaryotic genomes J Bacteriol 2000 182 18 5238 5250 10.1128/JB.182.18.5238-5250.2000 10960111 PMC94675

[B25] Urrutia AO Hurst LD Codon usage bias covaries with expression breadth and the rate of synonymous evolution in humans, but this is not evidence for selection Genetics 2001 159 3 1191 1199 11729162 10.1093/genetics/159.3.1191 PMC1461876

[B26] Novembre JA Accounting for background nucleotide composition when measuring codon usage bias Mol Biol Evol 2002 19 8 1390 1394 12140252 10.1093/oxfordjournals.molbev.a004201

[B27] Carbone A Zinovyev A Képès F Codon adaptation index as a measure of dominating codon bias Bioinformatics 2003 19 16 2005 2015 10.1093/bioinformatics/btg272 14594704

[B28] Kloster M Tang C SCUMBLE: a method for systematic and accurate detection of codon usage bias by maximum likelihood estimation Nucleic Acids Res 2008 36 11 3819 3827 10.1093/nar/gkn288 18495752 PMC2441815

[B29] Coghlan A Wolfe KH Relationship of codon bias to mRNA concentration and protein length in *Saccharomyces cerevisiae* Yeast 2000 16 12 1131 1145 10.1002/1097-0061(20000915)16:12<1131::AID-YEA609>3.0.CO;2-F 10953085

[B30] Supek F Vlahovicek K Comparison of codon usage measures and their applicability in prediction of microbial gene expressivity BMC Bioinformatics 2005 6 1 182 10.1186/1471-2105-6-182 16029499 PMC1199580

[B31] Merkl R A survey of codon and amino acid frequency bias in microbial genomes focusing on translational efficiency J Mol Evol 2003 57 453 466 10.1007/s00239-003-2499-1 14708578

[B32] Pruitt KD Tatusova T Klimke W Maglott DR NCBI Reference Sequences: current status, policy and new initiatives Nucleic Acids Res 2009 37 Database D32 36 10.1093/nar/gkn721 18927115 PMC2686572

[B33] Makarova KS Sorokin AV Novichkov PS Wolf YI Koonin EV Clusters of orthologous genes for 41 archaeal genomes and implications for evolutionary genomics of archaea Biol Direct 2007 2 33 10.1186/1745-6150-2-33 18042280 PMC2222616

[B34] dos Reis M Savva R Wernisch L Solving the riddle of codon usage preferences: a test for translational selection Nucleic Acids Res 2004 32 17 5036 5044 10.1093/nar/gkh834 15448185 PMC521650

[B35] Carbone A Kepes F Zinovyev A Codon bias signatures, organization of microorganisms in codon space, and lifestyle Mol Biol Evol 2005 22 3 547 561 10.1093/molbev/msi040 15537809

[B36] Ashburner M Ball CA Blake JA Botstein D Butler H Cherry JM Davis AP Dolinski K Dwight SS Eppig JT Gene ontology: tool for the unification of biology. The Gene Ontology Consortium Nat Genet 2000 25 1 25 29 10.1038/75556 10802651 PMC3037419

[B37] Ruepp A Zollner A Maier D Albermann K Hani J Mokrejs M Tetko I Güldener U Mannhaupt G Münsterkötter M The FunCat, a functional annotation scheme for systematic classification of proteins from whole genomes Nucleic Acids Res 2004 32 18 5539 5545 10.1093/nar/gkh894 15486203 PMC524302

[B38] Merkl R SIGI: score-based identification of genomic islands BMC Bioinformatics 2004 5 22 10.1186/1471-2105-5-22 15113412 PMC394314

[B39] Tatusov RL Fedorova ND Jackson JD Jacobs AR Kiryutin B Koonin EV Krylov DM Mazumder R Mekhedov SL Nikolskaya AN The COG database: an updated version includes Eukaryotes BMC Bioinformatics 2003 4 1 41 10.1186/1471-2105-4-41 12969510 PMC222959

[B40] Muller J Szklarczyk D Julien P Letunic I Roth A Kuhn M Powell S von Mering C Doerks T Jensen LJ eggNOG v2.0: extending the evolutionary genealogy of genes with enhanced non-supervised orthologous groups, species and functional annotations Nucleic Acids Res 2010 38 Database D190 195 10.1093/nar/gkp951 19900971 PMC2808932

[B41] Kanehisa M Goto S Furumichi M Tanabe M Hirakawa M KEGG for representation and analysis of molecular networks involving diseases and drugs Nucleic Acids Res 2010 38 Database D355 360 10.1093/nar/gkp896 19880382 PMC2808910

[B42] Galagan JE Nusbaum C Roy A Endrizzi MG Macdonald P FitzHugh W Calvo S Engels R Smirnov S Atnoor D The Genome of *M. acetivorans *Reveals Extensive Metabolic and Physiological Diversity Genome Res 2002 12 4 532 542 10.1101/gr.223902 11932238 PMC187521

[B43] Koch AL Microbial physiology and ecology of slow growth Microbiol Mol Biol Rev 1997 61 3 305 318 9293184 10.1128/mmbr.61.3.305-318.1997 PMC232613

[B44] Woese CR Olsen GJ Ibba M Soll D Aminoacyl-tRNA synthetases, the genetic code, and the evolutionary process Microbiol Mol Biol Rev 2000 64 1 202 236 10.1128/MMBR.64.1.202-236.2000 10704480 PMC98992

[B45] Brüggemann H Bäumer S Fricke WF Wiezer A Liesegang H Decker I Herzberg C Martinez-Arias R Merkl R Henne A The genome sequence of *Clostridium tetani*, the causative agent of tetanus disease Proc Natl Acad Sci USA 2003 100 3 1316 1321 10.1073/pnas.0335853100 12552129 PMC298770

[B46] Altschul SF Amino acid substitution matrices from an information theoretic perspective J Mol Biol 1991 219 3 555 565 10.1016/0022-2836(91)90193-A 2051488 PMC7130686

[B47] Henikoff S Henikoff JG Automated assembly of protein blocks for database searching Nucleic Acids Res 1991 19 23 6565 6572 10.1093/nar/19.23.6565 1754394 PMC329220

[B48] Waack S Keller O Asper R Brodag T Damm C Fricke W Surovcik K Meinicke P Merkl R Score-based prediction of genomic islands in prokaryotic genomes using hidden Markov models BMC Bioinformatics 2006 7 142 10.1186/1471-2105-7-142 16542435 PMC1489950

[B49] Hiraoka Y Kawamata K Haraguchi T Chikashige Y Codon usage bias is correlated with gene expression levels in the fission yeast *Schizosaccharomyces pombe* Genes Cells 2009 14 4 499 509 10.1111/j.1365-2443.2009.01284.x 19335619

[B50] UniProt Interface http://www.uniprot.org/jobs

