# Supplementary material for: Genes optimized by evolution for accurate and fast translation encode in Archaea and Bacteria a broad and characteristic spectrum of protein functions
Source: BMC Genomics. 2010 Nov 4;11:617. doi: 10.1186/1471-2164-11-617 (PMC3091758; doi:10.1186/1471-2164-11-617)
Supplement: Additional file 1 — Tables S1 - S4. [file 1471-2164-11-617-S1.PDF]

Additional file1, Table S1 – The composition of the dataset MG_CUB

Species	Super-kingdom	Temperature Range	Habitat	Oxygen requirement	Salinity	Optimal temperature	HSRG	GC-cont	$\overline{GCB_{Eff}}$	# Genes	# Genes Effectome
Acholeplasma laidlawii PG-8A	Bacteria	Mesophilic	Specialized	Facultative	NonHalophilic	37	6	0.32	0.06	1380	66
Acidobacteria bacterium Ellin345	Bacteria	Mesophilic	Terrestrial	Aerobic	Unknown	-	7	0.59	0.03	4777	78
Acidothermus cellulolyticus 11B	Bacteria	Thermophilic	Aquatic	Aerobic	Unknown	58	6	0.67	0.03	2157	75
Acidovorax avenae subsp. citrulli AAC00-1	Bacteria	Mesophilic	Multiple	Aerobic	NonHalophilic	-	14	0.69	0.07	4709	95
Acinetobacter baumannii AB0057	Bacteria	Mesophilic	Multiple	Aerobic	Unknown	-	8	0.4	0.14	3790	107
Acinetobacter sp. ADP1	Bacteria	Mesophilic	Multiple	Aerobic	NonHalophilic	37	12	0.42	0.16	3307	80
Actinobacillus pleuropneumoniae serovar 7 str. AP76	Bacteria	Mesophilic	Host_Associated	Facultative	Unknown	-	12	0.42	0.14	2131	98
Actinobacillus succinogenes 130Z	Bacteria	Mesophilic	Host_Associated	Unknown	NonHalophilic	37	17	0.46	0.16	2079	80
Aeromonas hydrophila subsp. hydrophila ATCC 7966	Bacteria	Mesophilic	Multiple	Facultative	Unknown	22-28	17	0.63	0.21	4122	130
Aeromonas salmonicida subsp. salmonicida A449	Bacteria	Mesophilic	Aquatic	Facultative	Unknown	-	15	0.6	0.17	4086	124
Agrobacterium radiobacter K84	Bacteria	Mesophilic	Terrestrial	Aerobic	NonHalophilic	25-28	14	0.61	0.11	3744	102
Agrobacterium tumefaciens str. C58	Bacteria	Mesophilic	Multiple	Aerobic	Unknown	25-28	11	0.6	0.11	2765	94
Agrobacterium vitis S4	Bacteria	Mesophilic	Multiple	Aerobic	Unknown	-	11	0.59	0.11	3234	88
Akkermansia muciniphila ATCC BAA-835	Bacteria	Mesophilic	Host_Associated	Anaerobic	Unknown	37	14	0.57	0.07	2138	88
Alcanivorax borkumensis SK2	Bacteria	Mesophilic	Aquatic	Aerobic	ModerateHalophilic	-	10	0.55	0.07	2755	89
Aliivibrio salmonicida LFI1238	Bacteria	Psychrophilic	Aquatic	Facultative	Unknown	-	9	0.4	0.15	2820	102
Alkaliphilus metalliredigens QYMF	Bacteria	Mesophilic	Unknown	Unknown	ModerateHalophilic	-	10	0.38	0.05	4625	128
Alkaliphilus oremlandii OhILAs	Bacteria	Mesophilic	Aquatic	Anaerobic	Unknown	-	8	0.37	0.11	2836	65
Alteromonas macleodii 'Deep ecotype'	Bacteria	Mesophilic	Aquatic	Aerobic	Unknown	-	8	0.46	0.13	4072	109
Anaeromyxobacter dehalogenans 2CP-1	Bacteria	Mesophilic	Terrestrial	Facultative	Unknown	-	8	0.75	0.02	4473	98
Anaeromyxobacter sp. K	Bacteria	Mesophilic	Terrestrial	Anaerobic	NonHalophilic	-	7	0.75	0.03	4457	119
Anaplasma marginale str. St. Maries	Bacteria	Unkown	Host_Associated	Aerobic	Unknown	-	13	0.5	0.03	948	82
Anaplasma phagocytophilum HZ	Bacteria	Mesophilic	Host_Associated	Aerobic	Unknown	37	7	0.43	0.04	1264	99
Anoxybacillus flavithermus WK1	Bacteria	Thermophilic	Specialized	Facultative	Unknown	60-65	14	0.42	0.12	2832	73
Archaeoglobus fulgidus DSM 4304	Archaea	Hyperthermophilic	Aquatic	Anaerobic	Unknown	83	6	0.49	0.03	2420	58
Arcobacter butzleri RM4018	Bacteria	Mesophilic	Multiple	Aerobic	Unknown	-	13	0.27	0.05	2259	91
Aromatoleum aromaticum EbN1	Bacteria	Mesophilic	Terrestrial	Facultative	NonHalophilic	26	10	0.66	0.03	4124	84
Arthrobacter aurescens TC1	Bacteria	Mesophilic	Terrestrial	Aerobic	NonHalophilic	30	17	0.63	0.11	4041	114
Arthrobacter chlorophenolicus A6	Bacteria	Mesophilic	Terrestrial	Aerobic	Unknown	-	19	0.67	0.10	3885	101

Arthrobacter sp. FB24	Bacteria	Mesophilic	Unknown	Unknown	Unknown	-	18	0.66	0.11	4146	104
Azorhizobium caulinodans ORS 571	Bacteria	Unkown	Host_Associated	Unknown	Unknown	-	10	0.68	0.04	4717	105
Bacillus amyloliquefaciens FZB42	Bacteria	Mesophilic	Terrestrial	Aerobic	Unknown	-	15	0.47	0.21	3693	90
Bacillus anthracis str. Ames	Bacteria	Mesophilic	Multiple	Facultative	Unknown	-	8	0.36	0.15	5311	126
Bacillus cereus ATCC 10987	Bacteria	Mesophilic	Terrestrial	Aerobic	Unknown	25-35	7	0.36	0.16	5603	124
Bacillus clausii KSM-K16	Bacteria	Unkown	Unknown	Unknown	Unknown	-	13	0.45	0.09	4096	78
Bacillus halodurans C-125	Bacteria	Mesophilic	Multiple	Facultative	Unknown	-	13	0.44	0.12	4066	93
Bacillus licheniformis ATCC 14580	Bacteria	Mesophilic	Terrestrial	Facultative	Unknown	-	13	0.47	0.21	4196	84
Bacillus pumilus SAFR-032	Bacteria	Mesophilic	Terrestrial	Aerobic	Unknown	-	14	0.42	0.16	3681	105
Bacillus subtilis subsp. subtilis str. 168	Bacteria	Mesophilic	Terrestrial	Facultative	Unknown	25-35	14	0.44	0.19	4105	99
Bacillus thuringiensis serovar konkukian str. 97-27	Bacteria	Mesophilic	Multiple	Facultative	Unknown	-	8	0.36	0.15	5117	123
Bacillus weihenstephanensis KBAB4	Bacteria	Mesophilic	Terrestrial	Aerobic	NonHalophilic	-	7	0.36	0.14	5155	132
Bacteroides fragilis YCH46	Bacteria	Mesophilic	Unknown	Anaerobic	Unknown	37	8	0.44	0.13	4578	70
Bacteroides thetaiotaomicron VPI-5482	Bacteria	Mesophilic	Host_Associated	Anaerobic	Unknown	-	7	0.44	0.12	4778	72
Bacteroides vulgatus ATCC 8482	Bacteria	Mesophilic	Host_Associated	Anaerobic	Unknown	-	5	0.43	0.11	4065	67
Bartonella bacilliformis KC583	Bacteria	Mesophilic	Host_Associated	Aerobic	Unknown	28	16	0.4	0.04	1283	70
Bartonella henselae str. Houston-1	Bacteria	Mesophilic	Host_Associated	Aerobic	Unknown	37	12	0.4	0.04	1488	44
Bartonella quintana str. Toulouse	Bacteria	Mesophilic	Host_Associated	Aerobic	Unknown	37	14	0.4	0.03	1142	66
Bartonella tribocorum CIP 105476	Bacteria	Mesophilic	Host_Associated	Aerobic	Unknown	-	13	0.41	0.04	2074	47
Bdellovibrio bacteriovorus HD100	Bacteria	Mesophilic	Multiple	Aerobic	Unknown	28-30	11	0.51	0.17	3587	143
Bifidobacterium adolescentis ATCC 15703	Bacteria	Mesophilic	Host_Associated	Anaerobic	NonHalophilic	37	10	0.6	0.12	1631	102
Bifidobacterium animalis subsp. lactis AD011	Bacteria	Mesophilic	Multiple	Anaerobic	NonHalophilic	39-40	11	0.62	0.10	1528	75
Bifidobacterium longum subsp. infantis ATCC 15697	Bacteria	Mesophilic	Host_Associated	Anaerobic	Unknown	37-41	8	0.61	0.11	2416	113
Bordetella avium 197N	Bacteria	Unkown	Host_Associated	Aerobic	Unknown	35-37	15	0.62	0.09	3381	97
Bordetella bronchiseptica RB50	Bacteria	Mesophilic	Host_Associated	Aerobic	Unknown	35-37	17	0.69	0.09	4994	100
Bordetella parapertussis 12822	Bacteria	Mesophilic	Host_Associated	Aerobic	Unknown	35-37	17	0.68	0.10	4185	91
Bordetella pertussis Tohama I	Bacteria	Mesophilic	Host_Associated	Aerobic	Unknown	35-37	17	0.68	0.10	3436	89
Bordetella petrii DSM 12804	Bacteria	Mesophilic	Aquatic	Anaerobic	Unknown	-	10	0.66	0.06	5027	114
Borrelia burgdorferi B31	Bacteria	Mesophilic	Host_Associated	Microaerophilic	Unknown	-	6	0.29	0.02	851	158
Borrelia duttonii Ly	Bacteria	Psychrophilic	Host_Associated	Aerobic	Unknown	-	7	0.28	0.02	820	257
Borrelia garinii PBi	Bacteria	Mesophilic	Host_Associated	Unknown	Unknown	-	8	0.29	0.02	832	129
Borrelia hermsii DAH	Bacteria	Mesophilic	Host_Associated	Aerobic	Unknown	-	7	0.3	0.02	819	189
Borrelia recurrentis A1	Bacteria	Mesophilic	Host_Associated	Aerobic	Unknown	-	7	0.28	0.02	800	270

Borrelia turicatae 91E135	Bacteria	Mesophilic	Host_Associated	Aerobic	Unknown	-	7	0.29	0.02	818	169
Bradyrhizobium japonicum USDA 110	Bacteria	Mesophilic	Host_Associated	Aerobic	Unknown	25-30	9	0.65	0.04	8317	167
Brucella abortus bv. 1 str. 9-941	Bacteria	Mesophilic	Host_Associated	Facultative	Unknown	37	15	0.58	0.09	2030	81
Brucella canis ATCC 23365	Bacteria	Unkown	Unknown	Unknown	Unknown	-	15	0.58	0.09	2102	84
Brucella melitensis 16M	Bacteria	Mesophilic	Host_Associated	Aerobic	Unknown	37	14	0.58	0.09	2059	80
Brucella ovis ATCC 25840	Bacteria	Mesophilic	Host_Associated	Facultative	Unknown	37	16	0.58	0.08	1928	79
Brucella suis 1330	Bacteria	Mesophilic	Host_Associated	Aerobic	Unknown	37	16	0.58	0.08	2123	82
Burkholderia ambifaria MC40-6	Bacteria	Mesophilic	Multiple	Unknown	NonHalophilic	-	15	0.68	0.12	3074	80
Burkholderia cenocepacia J2315	Bacteria	Unkown	Multiple	Facultative	Unknown	-	15	0.67	0.11	3464	99
Burkholderia multivorans ATCC 17616	Bacteria	Mesophilic	Host_Associated	Aerobic	NonHalophilic	-	14	0.68	0.11	3084	82
Burkholderia multivorans ATCC 17616	Bacteria	Mesophilic	Host_Associated	Aerobic	NonHalophilic	-	15	0.67	0.10	3146	80
Burkholderia phymatum STM815	Bacteria	Mesophilic	Host_Associated	Unknown	NonHalophilic	-	10	0.64	0.09	3072	96
Burkholderia phytofirmans PsJN	Bacteria	Mesophilic	Terrestrial	Aerobic	Unknown	30	12	0.63	0.09	3922	99
Burkholderia pseudomallei 1106a	Bacteria	Mesophilic	Terrestrial	Aerobic	Unknown	-	12	0.68	0.08	4015	109
Burkholderia sp. 383	Bacteria	Unkown	Multiple	Facultative	Unknown	-	11	0.67	0.11	3334	95
Burkholderia thailandensis E264	Bacteria	Mesophilic	Terrestrial	Aerobic	Unknown	25-42	12	0.68	0.07	3276	100
Burkholderia vietnamiensis G4	Bacteria	Unkown	Multiple	Facultative	Unknown	-	13	0.67	0.10	3274	85
Burkholderia xenovorans LB400	Bacteria	Mesophilic	Multiple	Aerobic	Unknown	30	13	0.63	0.10	4430	95
Campylobacter concisus 13826	Bacteria	Mesophilic	Host_Associated	Microaerophili c	Unknown	-	9	0.4	0.07	1929	134
Campylobacter curvus 525.92	Bacteria	Mesophilic	Host_Associated	Microaerophili c	Unknown	-	9	0.45	0.07	1931	107
Campylobacter fetus subsp. fetus 82-40	Bacteria	Unkown	Host_Associated	Microaerophili c	Unknown	-	11	0.34	0.05	1719	44
Campylobacter hominis ATCC BAA-381	Bacteria	Mesophilic	Host_Associated	Anaerobic	Unknown	37	7	0.33	0.04	1682	63
Campylobacter jejuni RM1221	Bacteria	Mesophilic	Multiple	Microaerophili c	Unknown	-	13	0.31	0.04	1838	63
Campylobacter lari RM2100	Bacteria	Mesophilic	Multiple	Microaerophili c	Unknown	-	9	0.3	0.04	1503	50
Candidatus Azobacteroides pseudotrichonymphae genomovar. CFP2	Bacteria	Mesophilic	Specialized	Unknown	Unknown	-	15	0.34	0.02	758	79
Candidatus Blochmannia floridanus	Bacteria	Mesophilic	Specialized	Unknown	Unknown	-	10	0.29	0.02	583	94
Candidatus Blochmannia pennsylvanicus str. BPEN	Bacteria	Unkown	Unknown	Unknown	Unknown	-	6	0.32	0.02	610	45
Candidatus Korarchaeum cryptofilum OPF8	Archaea	Thermophilic	Specialized	Anaerobic	Unknown	-	5	0.5	0.02	1602	65
Candidatus Methanoregula boonei 6A8	Archaea	Mesophilic	Terrestrial	Anaerobic	Unknown	37	7	0.56	0.06	2450	54
Candidatus Methanosphaerula palustris E1-9c	Archaea	Mesophilic	Specialized	Anaerobic	Unknown	30	5	0.57	0.05	2655	72
Candidatus Protochlamydia amoebophila UWE25	Bacteria	Mesophilic	Host_Associated	Unknown	Unknown	-	10	0.36	0.03	2031	100

Candidatus Ruthia magnifica str. Cm (Calyptogena magnifica)	Bacteria	Unkown	Unknown	Unknown	Unknown	-	10	0.35	0.03	976	39
Caulobacter crescentus NA1000	Bacteria	Mesophilic	Aquatic	Aerobic	Unknown	-	14	0.68	0.08	3876	103
Caulobacter sp. K31	Bacteria	Mesophilic	Unknown	Aerobic	NonHalophilic	-	17	0.68	0.06	5061	134
Cellvibrio japonicus Ueda107	Bacteria	Mesophilic	Terrestrial	Aerobic	Unknown	-	16	0.53	0.09	3754	90
Chlamydia muridarum Nigg	Bacteria	Mesophilic	Host_Associated	Unknown	Unknown	37	13	0.41	0.03	904	47
Chlamydia trachomatis 434/Bu	Bacteria	Mesophilic	Host_Associated	Unknown	Unknown	-	12	0.42	0.03	874	42
Chlamydia trachomatis A/HAR-13	Bacteria	Mesophilic	Host_Associated	Unknown	Unknown	-	13	0.42	0.03	911	47
Chlamydophila caviae GPIC	Bacteria	Mesophilic	Host_Associated	Unknown	Unknown	37	7	0.4	0.03	998	41
Chlamydophila felis Fe/C-56	Bacteria	Mesophilic	Host_Associated	Unknown	Unknown	37	6	0.4	0.03	1005	39
Chlorobium chlorochromatii CaD3	Bacteria	Mesophilic	Aquatic	Anaerobic	Unknown	-	9	0.45	0.04	2002	70
Chromobacterium violaceum ATCC 12472	Bacteria	Mesophilic	Multiple	Facultative	Unknown	25	15	0.66	0.13	4407	161
Chromohalobacter salexigens DSM 3043	Bacteria	Mesophilic	Aquatic	Facultative	ModerateHalophilic	37	13	0.65	0.07	3298	84
Citrobacter koseri ATCC BAA-895	Bacteria	Mesophilic	Multiple	Unknown	Unknown	-	11	0.55	0.17	4980	114
Clavibacter michiganensis subsp. michiganensis NCPPB 382	Bacteria	Mesophilic	Multiple	Aerobic	Unknown	25-28	12	0.73	0.03	2984	128
Clostridium acetobutylicum ATCC 824	Bacteria	Mesophilic	Multiple	Anaerobic	NonHalophilic	Oct-65	8	0.32	0.06	3672	50
Clostridium beijerinckii NCIMB 8052	Bacteria	Mesophilic	Unknown	Unknown	NonHalophilic	-	7	0.31	0.09	5020	70
Clostridium botulinum B1 str. Okra	Bacteria	Mesophilic	Multiple	Anaerobic	Unknown	37	8	0.29	0.09	3657	69
Clostridium cellulolyticum H10	Bacteria	Mesophilic	Terrestrial	Anaerobic	Unknown	-	12	0.38	0.07	3390	62
Clostridium difficile 630	Bacteria	Mesophilic	Multiple	Anaerobic	NonHalophilic	37	13	0.3	0.07	3742	106
Clostridium perfringens ATCC 13124	Bacteria	Mesophilic	Multiple	Anaerobic	NonHalophilic	37	7	0.29	0.12	2876	96
Clostridium phytofermentans ISDg	Bacteria	Mesophilic	Terrestrial	Anaerobic	Unknown	37	13	0.36	0.10	3902	100
Clostridium tetani E88	Bacteria	Mesophilic	Multiple	Anaerobic	NonHalophilic	37	5	0.29	0.06	2373	28
Clostridium thermocellum ATCC 27405	Bacteria	Thermophilic	Multiple	Anaerobic	Unknown	60	5	0.4	0.07	3189	30
Colwellia psychrerythraea 34H	Bacteria	Psychrophilic	Specialized	Facultative	Unknown	8	8	0.39	0.09	4910	105
Coprothermobacter proteolyticus DSM 5265	Bacteria	Thermophilic	Specialized	Anaerobic	Unknown	63	6	0.45	0.01	1482	89
Corynebacterium diphtheriae NCTC 13129	Bacteria	Mesophilic	Multiple	Aerobic	Unknown	37	15	0.54	0.16	2272	113
Corynebacterium efficiens YS-314	Bacteria	Mesophilic	Multiple	Facultative	Unknown	30-45	16	0.64	0.11	2938	118
Corynebacterium glutamicum ATCC 13032	Bacteria	Mesophilic	Multiple	Facultative	Unknown	30-40	9	0.55	0.14	2993	152
Corynebacterium glutamicum ATCC 13032	Bacteria	Mesophilic	Multiple	Facultative	Unknown	30-40	8	0.55	0.14	3057	158
Corynebacterium jeikeium K411	Bacteria	Mesophilic	Multiple	Facultative	NonHalophilic	-	8	0.62	0.13	2104	105
Corynebacterium urealyticum DSM 7109	Bacteria	Mesophilic	Host_Associated	Aerobic	Unknown	-	9	0.65	0.09	2024	121
Cronobacter sakazakii ATCC BAA-894	Bacteria	Mesophilic	Host_Associated	Anaerobic	NonHalophilic	37	8	0.58	0.21	4255	102
Cupriavidus taiwanensis	Bacteria	Mesophilic	Host_Associated	Facultative	Unknown	-	14	0.68	0.10	3135	103

Cytophaga hutchinsonii ATCC 33406	Bacteria	Mesophilic	Multiple	Aerobic	Unknown	30	9	0.4	0.09	3785	108
Dechloromonas aromatica RCB	Bacteria	Unkown	Multiple	Facultative	Unknown	-	6	0.6	0.09	4171	107
Dehalococcoides ethenogenes 195	Bacteria	Mesophilic	Multiple	Anaerobic	Unknown	35	8	0.5	0.04	1580	40
Dehalococcoides sp. CBDB1	Bacteria	Mesophilic	Multiple	Anaerobic	Unknown	-	9	0.48	0.04	1458	41
Deinococcus geothermalis DSM 11300	Bacteria	Mesophilic	Aquatic	Aerobic	Unknown	47	17	0.67	0.03	2330	80
Deinococcus radiodurans R1	Bacteria	Mesophilic	Terrestrial	Aerobic	Unknown	30-37	14	0.68	0.07	2629	113
Delftia acidovorans SPH-1	Bacteria	Mesophilic	Multiple	Aerobic	NonHalophilic	30	12	0.67	0.12	6040	110
Desulfitobacterium hafniense Y51	Bacteria	Mesophilic	Specialized	Anaerobic	Unknown	-	8	0.49	0.05	5060	97
Dictyoglomus turgidum DSM 6724	Bacteria	Thermophilic	Specialized	Anaerobic	Unknown	-	6	0.34	0.01	1744	109
Dinoroseobacter shibae DFL 12	Bacteria	Mesophilic	Unknown	Aerobic	Unknown	33	6	0.66	0.05	3577	97
Ehrlichia canis str. Jake	Bacteria	Unkown	Host_Associated	Unknown	Unknown	-	11	0.31	0.02	925	168
Ehrlichia chaffeensis str. Arkansas	Bacteria	Unkown	Host_Associated	Unknown	Unknown	-	8	0.32	0.03	1105	151
Ehrlichia ruminantium str. Gardel	Bacteria	Mesophilic	Host_Associated	Unknown	Unknown	-	11	0.31	0.02	950	124
Ehrlichia ruminantium str. Welgevonden	Bacteria	Mesophilic	Host_Associated	Unknown	Unknown	-	15	0.31	0.02	888	99
Ehrlichia ruminantium str. Welgevonden	Bacteria	Mesophilic	Host_Associated	Unknown	Unknown	-	12	0.31	0.03	958	93
Elusimicrobium minutum Pei191	Bacteria	Mesophilic	Host_Associated	Anaerobic	Unknown	-	10	0.41	0.06	1529	89
Enterobacter sp. 638	Bacteria	Unkown	Unknown	Unknown	Unknown	-	9	0.54	0.16	4115	120
Enterococcus faecalis V583	Bacteria	Mesophilic	Multiple	Facultative	Unknown	37	14	0.38	0.14	3113	92
Erwinia tasmaniensis Et1/99	Bacteria	Mesophilic	Host_Associated	Facultative	Unknown	-	11	0.55	0.15	3427	90
Erythrobacter litoralis HTCC2594	Bacteria	Unkown	Aquatic	Aerobic	Unknown	-	8	0.64	0.06	3011	87
Escherichia coli CFT073	Bacteria	Mesophilic	Host_Associated	Facultative	Unknown	37	10	0.52	0.16	5339	99
Exiguobacterium sibiricum 255-15	Bacteria	Psychrophilic	Specialized	Facultative	Unknown	-	13	0.49	0.23	3007	101
Finegoldia magna ATCC 29328	Bacteria	Mesophilic	Multiple	Anaerobic	NonHalophilic	-	5	0.33	0.07	1631	87
Flavobacterium johnsoniae UW101	Bacteria	Mesophilic	Multiple	Aerobic	NonHalophilic	20-30	7	0.35	0.08	5017	143
Flavobacterium psychrophilum JIP02/86	Bacteria	Psychrophilic	Aquatic	Aerobic	Unknown	-	14	0.33	0.06	2412	87
Francisella novicida U112	Bacteria	Mesophilic	Multiple	Aerobic	Unknown	-	14	0.33	0.07	1719	52
Francisella philomiragia subsp. philomiragia ATCC 25017	Bacteria	Mesophilic	Multiple	Aerobic	Unknown	-	12	0.33	0.06	1911	61
Francisella tularensis subsp. holarctica FTNF002-00	Bacteria	Mesophilic	Multiple	Aerobic	Unknown	-	13	0.33	0.06	1580	53
Fusobacterium nucleatum subsp. nucleatum ATCC 25586	Bacteria	Mesophilic	Host_Associated	Anaerobic	Unknown	37	11	0.27	0.04	2067	88
Geobacillus kaustophilus HTA426	Bacteria	Thermophilic	Aquatic	Aerobic	Unknown	-	13	0.53	0.05	3498	83
Geobacillus thermodenitrificans NG80-2	Bacteria	Thermophilic	Specialized	Facultative	NonHalophilic	65	11	0.5	0.08	3392	64
Gloeobacter violaceus PCC 7421	Bacteria	Mesophilic	Terrestrial	Unknown	Unknown	-	6	0.63	0.03	4430	145
Gluconacetobacter diazotrophicus PAl 5	Bacteria	Mesophilic	Host_Associated	Aerobic	Unknown	30	13	0.67	0.05	3472	82
Gluconobacter oxydans 621H	Bacteria	Mesophilic	Multiple	Aerobic	Unknown	25-30	13	0.62	0.09	2432	105

Gramella forsetii KT0803	Bacteria	Mesophilic	Multiple	Aerobic	NonHalophilic	-	10	0.37	0.05	3584	82
Granulibacter bethesdensis CGDNIH1	Bacteria	Mesophilic	Multiple	Unknown	NonHalophilic	-	10	0.6	0.07	2437	85
Haemophilus ducreyi 35000HP	Bacteria	Mesophilic	Host_Associated	Anaerobic	Unknown	35-37	14	0.39	0.09	1717	60
Haemophilus influenzae 86-028NP	Bacteria	Mesophilic	Host_Associated	Facultative	Unknown	35-37	12	0.39	0.12	1792	74
Haemophilus parasuis SH0165	Bacteria	Mesophilic	Host_Associated	Facultative	Unknown	-	10	0.41	0.13	2021	103
Haemophilus somnus 2336	Bacteria	Mesophilic	Host_Associated	Facultative	Unknown	35-37	17	0.38	0.07	1980	89
Haloarcula marismortui ATCC 43049	Archaea	Mesophilic	Aquatic	Aerobic	ExtremeHalophilic	40-50	10	0.63	0.03	3131	106
Halorubrum lacusprofundi ATCC 49239	Archaea	Mesophilic	Aquatic	Aerobic	ExtremeHalophilic	-	6	0.68	0.03	2711	67
Helicobacter acinonychis str. Sheeba	Bacteria	Mesophilic	Host_Associated	Microaerophilic	NonHalophilic	37	8	0.39	0.02	1612	62
Herminiimonas arsenicoxydans	Bacteria	Mesophilic	Aquatic	Anaerobic	NonHalophilic	-	11	0.55	0.10	3295	108
Herpetosiphon aurantiacus ATCC 23779	Bacteria	Mesophilic	Multiple	Aerobic	Unknown	-	7	0.51	0.10	4976	91
Hydrogenobaculum sp. Y04AAS1	Bacteria	Thermophilic	Aquatic	Aerobic	Unknown	58	7	0.35	0.03	1629	77
Hyphomonas neptunium ATCC 15444	Bacteria	Mesophilic	Aquatic	Aerobic	Unknown	37	7	0.62	0.06	3505	95
Idiomarina loihiensis L2TR	Bacteria	Mesophilic	Specialized	Aerobic	Unknown	Apr-46	7	0.48	0.09	2628	80
Ignicoccus hospitalis KIN4/I	Archaea	Hyperthermophilic	Aquatic	Anaerobic	Unknown	-	5	0.57	0.05	1434	33
Jannaschia sp. CCS1	Bacteria	Mesophilic	Aquatic	Aerobic	Unknown	30	5	0.63	0.06	4212	76
Janthinobacterium sp. Marseille	Bacteria	Mesophilic	Aquatic	Unknown	Unknown	-	8	0.55	0.13	3697	89
Kineococcus radiotolerans SRS30216	Bacteria	Mesophilic	Multiple	Aerobic	Unknown	32	11	0.75	0.03	4480	100
Klebsiella pneumoniae 342	Bacteria	Mesophilic	Host_Associated	Facultative	Unknown	-	11	0.59	0.22	5425	122
Klebsiella pneumoniae subsp. pneumoniae MGH 78578	Bacteria	Mesophilic	Multiple	Facultative	Unknown	37	12	0.59	0.22	4776	118
Kocuria rhizophila DC2201	Bacteria	Mesophilic	Multiple	Aerobic	Unknown	-	14	0.71	0.06	2357	97
Lactobacillus acidophilus NCFM	Bacteria	Mesophilic	Multiple	Facultative	Unknown	25-35	11	0.35	0.15	1862	80
Lactobacillus brevis ATCC 367	Bacteria	Mesophilic	Multiple	Facultative	Unknown	25-35	9	0.47	0.15	2185	82
Lactobacillus casei BL23	Bacteria	Mesophilic	Specialized	Facultative	Unknown	-	8	0.47	0.10	3044	77
Lactobacillus delbrueckii subsp. bulgaricus ATCC BAA-365	Bacteria	Mesophilic	Multiple	Facultative	Unknown	42	14	0.52	0.27	1721	84
Lactobacillus fermentum IFO 3956	Bacteria	Mesophilic	Multiple	Facultative	NonHalophilic	-	12	0.53	0.16	1843	87
Lactobacillus gasseri ATCC 33323	Bacteria	Mesophilic	Host_Associated	Facultative	Unknown	25-35	9	0.36	0.14	1755	62
Lactobacillus helveticus DPC 4571	Bacteria	Mesophilic	Multiple	Facultative	NonHalophilic	-	7	0.38	0.14	1610	102
Lactobacillus johnsonii NCC 533	Bacteria	Mesophilic	Host_Associated	Facultative	Unknown	25-35	10	0.35	0.17	1821	68
Lactobacillus plantarum WCFS1	Bacteria	Mesophilic	Host_Associated	Facultative	Unknown	25-35	10	0.46	0.14	3007	87
Lactobacillus reuteri DSM 20016	Bacteria	Mesophilic	Multiple	Facultative	Unknown	-	14	0.4	0.16	1900	81
Lactobacillus sakei subsp. sakei 23K	Bacteria	Mesophilic	Multiple	Facultative	Unknown	-	8	0.42	0.14	1879	104
Lactobacillus salivarius UCC118	Bacteria	Mesophilic	Host_Associated	Facultative	Unknown	-	12	0.33	0.13	1717	88

Lactococcus lactis subsp. cremoris MG1363	Bacteria	Mesophilic	Multiple	Facultative	NonHalophilic	40	12	0.37	0.22	2434	84
Legionella pneumophila str. Corby	Bacteria	Mesophilic	Host_Associated	Aerobic	Unknown	-	9	0.39	0.04	3206	63
Leifsonia xyli subsp. xyli str. CTCB07	Bacteria	Mesophilic	Host_Associated	Aerobic	Unknown	20-25	17	0.68	0.05	2030	80
Leptospira borgpetersenii serovar Hardjo-bovis L550	Bacteria	Mesophilic	Host_Associated	Aerobic	NonHalophilic	-	12	0.41	0.04	2703	75
Leuconostoc citreum KM20	Bacteria	Mesophilic	Unknown	Facultative	NonHalophilic	-	14	0.4	0.20	1702	72
Leuconostoc mesenteroides subsp. mesenteroides ATCC 8293	Bacteria	Mesophilic	Multiple	Facultative	Unknown	20-30	14	0.38	0.18	1970	70
Listeria innocua Clip11262	Bacteria	Mesophilic	Multiple	Facultative	Unknown	30-37	9	0.38	0.09	2968	118
Listeria monocytogenes HCC23	Bacteria	Mesophilic	Host_Associated	Facultative	Unknown	30-37	9	0.39	0.09	2974	112
Listeria welshimeri serovar 6b str. SLCC5334	Bacteria	Mesophilic	Multiple	Facultative	Unknown	-	9	0.37	0.09	2774	98
Lysinibacillus sphaericus C3-41	Bacteria	Mesophilic	Specialized	Aerobic	Unknown	-	13	0.38	0.16	4584	102
Macrococcus caseolyticus JCSC5402	Bacteria	Mesophilic	Multiple	Facultative	Unknown	35	17	0.38	0.13	1950	71
Magnetospirillum magneticum AMB-1	Bacteria	Mesophilic	Aquatic	Microaerophilic	Unknown	-	13	0.66	0.05	4559	159
Mannheimia succiniciproducens MBEL55E	Bacteria	Mesophilic	Host_Associated	Anaerobic	NonHalophilic	37	10	0.44	0.15	2369	80
Maricaulis maris MCS10	Bacteria	Mesophilic	Aquatic	Facultative	Unknown	20-25	7	0.63	0.05	3063	42
Marinobacter aquaeolei VT8	Bacteria	Mesophilic	Aquatic	Facultative	ModerateHalophilic	30	10	0.58	0.11	3858	94
Marinomonas sp. MWYL1	Bacteria	Mesophilic	Aquatic	Aerobic	Unknown	-	10	0.43	0.12	4439	105
Mesoplasma florum L1	Bacteria	Mesophilic	Host_Associated	Facultative	Unknown	20-40	11	0.27	0.07	682	68
Mesorhizobium loti MAFF303099	Bacteria	Mesophilic	Multiple	Aerobic	Unknown	-	14	0.64	0.05	6743	130
Mesorhizobium sp. BNC1	Bacteria	Unkown	Multiple	Aerobic	Unknown	-	7	0.62	0.03	4064	110
Methanobrevibacter smithii ATCC 35061	Archaea	Mesophilic	Multiple	Anaerobic	Unknown	37-40	6	0.32	0.08	1793	72
Methanocaldococcus jannaschii DSM 2661	Archaea	Hyperthermophilic	Aquatic	Anaerobic	ModerateHalophilic	85	11	0.32	0.07	1729	53
Methanococcus aeolicus Nankai-3	Archaea	Mesophilic	Aquatic	Anaerobic	Unknown	42	10	0.31	0.07	1490	59
Methanococcus vannielii SB	Archaea	Mesophilic	Aquatic	Anaerobic	NonHalophilic	30	9	0.32	0.09	1678	76
Methanopyrus kandleri AV19	Archaea	Hyperthermophilic	Specialized	Anaerobic	ModerateHalophilic	98	7	0.61	0.04	1687	68
Methanosarcina mazei Go1	Archaea	Mesophilic	Multiple	Anaerobic	Unknown	30-40	5	0.44	0.08	3370	47
Methylobacterium chloromethanicum CM4	Bacteria	Mesophilic	Terrestrial	Aerobic	Unknown	30	9	0.69	0.04	5173	130
Methylobacterium extorquens PA1	Bacteria	Mesophilic	Multiple	Facultative	Unknown	25-30	10	0.69	0.05	4829	83
Methylobacterium nodulans ORS 2060	Bacteria	Mesophilic	Host_Associated	Aerobic	Unknown	-	14	0.7	0.02	7355	241
Methylobacterium populi BJ001	Bacteria	Mesophilic	Unknown	Aerobic	NonHalophilic	20-30	12	0.7	0.04	5314	134
Methylobacterium radiotolerans JCM 2831	Bacteria	Mesophilic	Host_Associated	Aerobic	NonHalophilic	-	5	0.72	0.05	5686	90
Methylobacterium sp. 4-46	Bacteria	Mesophilic	Multiple	Facultative	Unknown	-	12	0.72	0.02	6609	198
Methylocella silvestris BL2	Bacteria	Mesophilic	Terrestrial	Aerobic	Unknown	-	7	0.64	0.04	3818	59
Mycobacterium avium 104	Bacteria	Mesophilic	Host_Associated	Aerobic	Unknown	37	6	0.69	0.03	5120	87

Mycobacterium avium subsp. paratuberculosis K-10	Bacteria	Mesophilic	Multiple	Aerobic	Unknown	37	6	0.7	0.03	4350	60
Mycobacterium bovis BCG str. Pasteur 1173P2	Bacteria	Mesophilic	Host_Associated	Aerobic	NonHalophilic	-	6	0.66	0.02	3952	73
Mycobacterium gilvum PYR-GCK	Bacteria	Mesophilic	Unknown	Unknown	Unknown	-	8	0.68	0.04	5241	102
Mycobacterium leprae TN	Bacteria	Mesophilic	Host_Associated	Aerobic	NonHalophilic	37	11	0.6	0.02	1605	42
Mycobacterium smegmatis str. MC2 155	Bacteria	Mesophilic	Host_Associated	Aerobic	Unknown	37	9	0.68	0.04	6716	88
Mycobacterium sp. JLS	Bacteria	Mesophilic	Multiple	Unknown	NonHalophilic	-	12	0.69	0.03	5739	104
Mycobacterium tuberculosis H37Ra	Bacteria	Unkown	Host_Associated	Aerobic	NonHalophilic	37	6	0.66	0.02	4034	72
Mycobacterium ulcerans Agy99	Bacteria	Mesophilic	Host_Associated	Aerobic	NonHalophilic	32	5	0.66	0.05	4160	62
Mycobacterium vanbaalenii PYR-1	Bacteria	Mesophilic	Unknown	Aerobic	Unknown	24-37	10	0.68	0.04	5979	119
Mycoplasma agalactiae PG2	Bacteria	Psychrophilic	Host_Associated	Facultative	Unknown	-	15	0.3	0.07	742	51
Mycoplasma arthritidis 158L3-1	Bacteria	Mesophilic	Host_Associated	Facultative	NonHalophilic	37	13	0.31	0.05	631	44
Mycoplasma capricolum subsp. capricolum ATCC 27343	Bacteria	Mesophilic	Host_Associated	Facultative	NonHalophilic	37	15	0.24	0.04	812	44
Mycoplasma gallisepticum R	Bacteria	Mesophilic	Host_Associated	Facultative	NonHalophilic	37	7	0.32	0.05	726	31
Mycoplasma genitalium G37	Bacteria	Mesophilic	Host_Associated	Facultative	NonHalophilic	37	8	0.32	0.03	476	59
Mycoplasma mobile 163K	Bacteria	Mesophilic	Host_Associated	Facultative	NonHalophilic	20	13	0.25	0.03	633	35
Mycoplasma mycoides subsp. mycoides SC str. PG1	Bacteria	Mesophilic	Host_Associated	Facultative	NonHalophilic	37	14	0.24	0.04	1016	56
Mycoplasma penetrans HF-2	Bacteria	Mesophilic	Host_Associated	Facultative	NonHalophilic	37	8	0.26	0.03	1037	50
Mycoplasma pulmonis UAB CTIP	Bacteria	Mesophilic	Host_Associated	Facultative	NonHalophilic	37	10	0.27	0.03	782	37
Mycoplasma synoviae 53	Bacteria	Mesophilic	Host_Associated	Facultative	NonHalophilic	37	13	0.29	0.05	659	31
Nautilia profundicola AmH	Bacteria	Mesophilic	Multiple	Anaerobic	Unknown	45	7	0.34	0.09	1730	87
Neisseria gonorrhoeae NCCP11945	Bacteria	Mesophilic	Host_Associated	Aerobic	Unknown	35-37	15	0.54	0.10	2662	147
Neorickettsia sennetsu str. Miyayama	Bacteria	Unkown	Multiple	Unknown	Unknown	-	13	0.41	0.03	932	111
Nitratiruptor sp. SB155-2	Bacteria	Thermophilic	Specialized	Anaerobic	Unknown	-	9	0.4	0.05	1843	65
Nitrobacter hamburgensis X14	Bacteria	Mesophilic	Terrestrial	Aerobic	Unknown	-	5	0.63	0.03	3804	132
Nitrosomonas europaea ATCC 19718	Bacteria	Mesophilic	Multiple	Aerobic	Unknown	-	13	0.52	0.09	2461	123
Nitrosomonas eutropha C91	Bacteria	Unkown	Multiple	Unknown	Unknown	-	18	0.49	0.07	2444	129
Nitrosopumilus maritimus SCM1	Archaea	Mesophilic	Aquatic	Aerobic	Unknown	-	6	0.35	0.04	1795	52
Nocardia farcinica IFM 10152	Bacteria	Mesophilic	Multiple	Aerobic	NonHalophilic	37	9	0.71	0.04	5683	114
Nocardioides sp. JS614	Bacteria	Mesophilic	Terrestrial	Aerobic	NonHalophilic	30	16	0.72	0.03	4645	116
Novosphingobium aromaticivorans DSM 12444	Bacteria	Unkown	Multiple	Aerobic	Unknown	-	13	0.66	0.07	3324	116
Oceanobacillus iheyensis HTE831	Bacteria	Mesophilic	Multiple	Aerobic	ModerateHalophilic	30	15	0.36	0.10	3500	91
Ochrobactrum anthropi ATCC 49188	Bacteria	Mesophilic	Terrestrial	Unknown	NonHalophilic	-	15	0.57	0.09	2731	106
Oenococcus oeni PSU-1	Bacteria	Mesophilic	Multiple	Facultative	Unknown	17-25	15	0.39	0.05	1691	67
Oligotropha carboxidovorans OM5	Bacteria	Mesophilic	Multiple	Unknown	Unknown	-	10	0.63	0.04	3722	131

Opitutus terrae PB90-1	Bacteria	Mesophilic	Aquatic	Anaerobic	Unknown	-	8	0.66	0.04	4612	202
Orientia tsutsugamushi str. Ikeda	Bacteria	Mesophilic	Host_Associated	Unknown	NonHalophilic	-	10	0.32	0.03	1967	57
Parabacteroides distasonis ATCC 8503	Bacteria	Mesophilic	Host_Associated	Anaerobic	Unknown	-	6	0.46	0.11	3850	81
Paracoccus denitrificans PD1222	Bacteria	Mesophilic	Multiple	Aerobic	Unknown	25-30	13	0.67	0.04	2799	81
Paracoccus denitrificans PD1222	Bacteria	Mesophilic	Multiple	Aerobic	Unknown	25-30	9	0.67	0.04	1662	61
Pasteurella multocida subsp. multocida str. Pm70	Bacteria	Mesophilic	Host_Associated	Facultative	Unknown	37	12	0.41	0.11	2015	72
Pectobacterium atrosepticum SCRI1043	Bacteria	Mesophilic	Multiple	Facultative	NonHalophilic	27-30	10	0.52	0.12	4472	108
Pediococcus pentosaceus ATCC 25745	Bacteria	Mesophilic	Multiple	Facultative	Unknown	30	10	0.38	0.16	1755	73
Pelodictyon luteolum DSM 273	Bacteria	Mesophilic	Multiple	Anaerobic	Unknown	25	5	0.58	0.04	2083	60
Photobacterium profundum SS9	Bacteria	Psychrophilic	Multiple	Facultative	NonHalophilic	15	7	0.43	0.15	3416	105
Photorhabdus luminescens subsp. laumondii TTO1	Bacteria	Mesophilic	Host_Associated	Facultative	Unknown	-	15	0.44	0.11	4683	89
Polaromonas sp. JS666	Bacteria	Mesophilic	Multiple	Aerobic	NonHalophilic	20	9	0.63	0.07	4817	103
Polynucleobacter necessarius subsp. asymbioticus QLW-P1DMWA-1	Bacteria	Mesophilic	Aquatic	Aerobic	Unknown	-	7	0.45	0.10	2077	77
Porphyromonas gingivalis ATCC 33277	Bacteria	Mesophilic	Host_Associated	Anaerobic	Unknown	37	7	0.5	0.05	2090	101
Propionibacterium acnes KPA171202	Bacteria	Mesophilic	Host_Associated	Anaerobic	NonHalophilic	37	14	0.6	0.08	2297	91
Pseudoalteromonas atlantica T6c	Bacteria	Mesophilic	Aquatic	Aerobic	Unknown	-	11	0.45	0.12	4281	96
Pseudoalteromonas haloplanktis TAC125	Bacteria	Psychrophilic	Aquatic	Aerobic	Unknown	-	12	0.41	0.12	2939	93
Pseudomonas aeruginosa PA7	Bacteria	Mesophilic	Multiple	Aerobic	Unknown	25-30	12	0.67	0.12	6286	177
Pseudomonas entomophila L48	Bacteria	Unkown	Multiple	Unknown	Unknown	-	12	0.65	0.17	5134	105
Pseudomonas fluorescens Pf-5	Bacteria	Mesophilic	Multiple	Aerobic	Unknown	25-30	10	0.64	0.18	6138	126
Pseudomonas mendocina ymp	Bacteria	Unkown	Multiple	Aerobic	NonHalophilic	-	12	0.65	0.14	4594	107
Pseudomonas putida GB-1	Bacteria	Mesophilic	Multiple	Aerobic	NonHalophilic	-	12	0.63	0.13	5409	119
Pseudomonas putida KT2440	Bacteria	Mesophilic	Multiple	Aerobic	Unknown	-	10	0.62	0.13	5350	116
Pseudomonas stutzeri A1501	Bacteria	Mesophilic	Host_Associated	Aerobic	Unknown	-	13	0.64	0.10	4128	100
Pseudomonas syringae pv. tomato str. DC3000	Bacteria	Mesophilic	Multiple	Aerobic	Unknown	-	8	0.59	0.11	5476	112
Psychrobacter arcticus 273-4	Bacteria	Psychrophilic	Specialized	Unknown	Unknown	22	7	0.45	0.10	2120	79
Psychrobacter cryohalolentis K5	Bacteria	Psychrophilic	Multiple	Unknown	ModerateHalophilic	-	7	0.44	0.09	2467	78
Psychrobacter sp. PRwf-1	Bacteria	Mesophilic	Aquatic	Aerobic	Unknown	Apr-37	8	0.46	0.16	2370	96
Psychromonas ingrahamii 37	Bacteria	Psychrophilic	Aquatic	Anaerobic	Unknown	-	11	0.41	0.13	3545	94
Pyrococcus abyssi GE5	Archaea	Hyperthermophilic	Aquatic	Anaerobic	Unknown	103	7	0.45	0.07	1780	30
Ralstonia eutropha H16	Bacteria	Mesophilic	Specialized	Facultative	NonHalophilic	30	13	0.67	0.08	3651	111
Ralstonia pickettii 12J	Bacteria	Mesophilic	Multiple	Aerobic	NonHalophilic	-	11	0.64	0.07	3709	111
Ralstonia solanacearum GMI1000	Bacteria	Mesophilic	Multiple	Aerobic	Unknown	-	15	0.68	0.09	3437	94

Renibacterium salmoninarum ATCC 33209	Bacteria	Mesophilic	Host_Associated	Facultative	Unknown	15	17	0.57	0.11	3507	85
Rhizobium etli CIAT 652	Bacteria	Mesophilic	Host_Associated	Aerobic	Unknown	-	8	0.63	0.10	4343	84
Rhizobium leguminosarum bv. viciae 3841	Bacteria	Mesophilic	Host_Associated	Aerobic	Unknown	25-30	8	0.62	0.08	4694	98
Rhodobacter sphaeroides KD131	Bacteria	Mesophilic	Multiple	Facultative	NonHalophilic	25-35	7	0.69	0.06	3101	101
Rhodopirellula baltica SH 1	Bacteria	Mesophilic	Aquatic	Aerobic	Unknown	28	8	0.56	0.06	7325	128
Rhodopseudomonas palustris TIE-1	Bacteria	Mesophilic	Unknown	Facultative	Unknown	25-30 C	12	0.65	0.04	5246	155
Rhodospirillum centenum SW	Bacteria	Mesophilic	Aquatic	Facultative	Unknown	40-42	9	0.71	0.04	4002	84
Rhodospirillum rubrum ATCC 11170	Bacteria	Mesophilic	Multiple	Facultative	Unknown	25-30	9	0.66	0.05	3791	109
Rickettsia akari str. Hartford	Bacteria	Mesophilic	Host_Associated	Aerobic	Unknown	-	7	0.33	0.02	1259	127
Rickettsia bellii OSU 85-389	Bacteria	Unkown	Unknown	Unknown	Unknown	-	6	0.32	0.02	1476	114
Rickettsia conorii str. Malish 7	Bacteria	Mesophilic	Host_Associated	Aerobic	Unknown	-	11	0.33	0.02	1374	117
Rickettsia rickettsii str. Iowa	Bacteria	Mesophilic	Host_Associated	Aerobic	Unknown	-	5	0.33	0.02	1384	155
Roseobacter denitrificans OCh 114	Bacteria	Mesophilic	Multiple	Unknown	Unknown	-	11	0.6	0.05	3946	98
Ruegeria sp. TM1040	Bacteria	Unkown	Multiple	Unknown	Unknown	-	9	0.61	0.13	3030	89
Saccharophagus degradans 2-40	Bacteria	Mesophilic	Aquatic	Aerobic	Unknown	Apr-37	9	0.47	0.13	4007	91
Saccharopolyspora erythraea NRRL 2338	Bacteria	Mesophilic	Terrestrial	Aerobic	Unknown	25-32 (28)	7	0.72	0.03	7197	196
Salinispora arenicola CNS-205	Bacteria	Mesophilic	Aquatic	Aerobic	Unknown	-	10	0.7	0.04	4917	78
Salinispora tropica CNB-440	Bacteria	Mesophilic	Aquatic	Aerobic	Unknown	28	12	0.7	0.04	4536	73
Salmonella enterica subsp. enterica serovar Heidelberg str. SL476	Bacteria	Mesophilic	Multiple	Facultative	Unknown	-	12	0.53	0.17	4650	110
Salmonella enterica subsp. enterica serovar Newport str. SL254	Bacteria	Mesophilic	Multiple	Facultative	Unknown	-	12	0.54		0	0
Serratia proteamaculans 568	Bacteria	Mesophilic	Multiple	Facultative	NonHalophilic	-	8	0.56	0.15	4891	110
Shewanella amazonensis SB2B	Bacteria	Mesophilic	Multiple	Facultative	Unknown	37	11	0.54	0.19	3645	106
Shewanella baltica OS195	Bacteria	Mesophilic	Aquatic	Facultative	Unknown	-	14	0.47	0.15	4499	110
Shewanella denitrificans OS217	Bacteria	Mesophilic	Aquatic	Facultative	Unknown	20-25	12	0.46	0.17	3754	88
Shewanella frigidimarina NCIMB 400	Bacteria	Mesophilic	Multiple	Facultative	Unknown	20-22	10	0.43	0.11	4029	92
Shewanella halifaxensis HAW-EB4	Bacteria	Psychrophilic	Unknown	Facultative	Unknown	10	9	0.46	0.14	4278	112
Shewanella loihica PV-4	Bacteria	Mesophilic	Multiple	Facultative	Unknown	-	14	0.55	0.26	3859	117
Shewanella oneidensis MR-1	Bacteria	Mesophilic	Multiple	Facultative	Unknown	-	12	0.47	0.18	4318	93
Shewanella pealeana ATCC 700345	Bacteria	Mesophilic	Unknown	Facultative	Unknown	-	8	0.46	0.14	4241	121
Shewanella piezotolerans WP3	Bacteria	Mesophilic	Specialized	Facultative	Unknown	15-20	10	0.44	0.12	4933	97
Shewanella putrefaciens CN-32	Bacteria	Mesophilic	Multiple	Facultative	Unknown	-	11	0.45	0.14	3972	95
Shewanella sediminis HAW-EB3	Bacteria	Psychrophilic	Aquatic	Facultative	Unknown	10	12	0.47	0.16	4497	98
Shewanella sp. ANA-3	Bacteria	Mesophilic	Multiple	Facultative	Unknown	-	9	0.49	0.20	4111	106

Shewanella woodyi ATCC 51908	Bacteria	Mesophilic	Multiple	Facultative	NonHalophilic	25	10	0.45	0.14	4880	106
Shigella boydii CDC 3083-94	Bacteria	Mesophilic	Host_Associated	Facultative	NonHalophilic	37	6	0.53	0.16	4246	108
Shigella dysenteriae Sd197	Bacteria	Mesophilic	Host_Associated	Facultative	NonHalophilic	37	7	0.53	0.16	4271	110
Shigella flexneri 2a str. 301	Bacteria	Mesophilic	Host_Associated	Facultative	Unknown	37	7	0.52	0.15	4177	109
Shigella sonnei Ss046	Bacteria	Mesophilic	Host_Associated	Facultative	NonHalophilic	37	8	0.52	0.15	4219	113
Silicibacter pomeroyi DSS-3	Bacteria	Unkown	Aquatic	Aerobic	Unknown	-	14	0.65	0.07	3810	72
Sinorhizobium medicae WSM419	Bacteria	Mesophilic	Multiple	Aerobic	NonHalophilic	28	15	0.62	0.07	3529	78
Sinorhizobium meliloti 1021	Bacteria	Mesophilic	Multiple	Aerobic	Unknown	25-30	14	0.64	0.08	3359	95
Sodalis glossinidius str. 'morsitans'	Bacteria	Mesophilic	Host_Associated	Microaerophilic	NonHalophilic	25	16	0.56	0.12	2432	86
Sphingomonas wittichii RW1	Bacteria	Mesophilic	Aquatic	Aerobic	Unknown	-	11	0.69	0.05	4850	112
Sphingopyxis alaskensis RB2256	Bacteria	Unkown	Aquatic	Aerobic	Unknown	-	9	0.66	0.05	3165	85
Staphylococcus aureus subsp. aureus NCTC 8325	Bacteria	Mesophilic	Host_Associated	Facultative	Unknown	30-37	10	0.34	0.12	2892	89
Staphylococcus epidermidis RP62A	Bacteria	Mesophilic	Host_Associated	Facultative	Unknown	30-37	9	0.33	0.11	2494	90
Staphylococcus haemolyticus JCSC1435	Bacteria	Mesophilic	Host_Associated	Facultative	Unknown	30-37	10	0.34	0.11	2676	109
Staphylococcus saprophyticus subsp. saprophyticus ATCC 15305	Bacteria	Mesophilic	Host_Associated	Aerobic	Unknown	-	13	0.34	0.12	2446	95
Stenotrophomonas maltophilia K279a	Bacteria	Mesophilic	Multiple	Aerobic	Unknown	-	10	0.67	0.07	4386	118
Streptococcus agalactiae 2603V/R	Bacteria	Mesophilic	Host_Associated	Facultative	Unknown	37	15	0.36	0.18	2124	74
Streptococcus equi subsp. zooepidemicus MGCS10565	Bacteria	Mesophilic	Host_Associated	Facultative	Unknown	-	17	0.43	0.21	1893	70
Streptococcus gordonii str. Challis substr. CH1	Bacteria	Mesophilic	Host_Associated	Facultative	Unknown	37	11	0.41	0.22	2051	88
Streptococcus mutans UA159	Bacteria	Mesophilic	Host_Associated	Facultative	Unknown	37	15	0.38	0.12	1960	64
Streptococcus pneumoniae CGSP14	Bacteria	Mesophilic	Multiple	Facultative	Unknown	-	13	0.4	0.20	2206	100
Streptococcus pyogenes MGAS10270	Bacteria	Mesophilic	Host_Associated	Facultative	NonHalophilic	-	15	0.39	0.20	1986	71
Streptococcus sanguinis SK36	Bacteria	Mesophilic	Host_Associated	Facultative	Unknown	-	15	0.45	0.23	2270	85
Streptococcus suis 05ZYH33	Bacteria	Mesophilic	Multiple	Facultative	NonHalophilic	37	14	0.42	0.21	2186	96
Streptococcus thermophilus CNRZ1066	Bacteria	Thermophilic	Multiple	Anaerobic	Unknown	45	16	0.4	0.18	1915	82
Streptococcus uberis 0140J	Bacteria	Mesophilic	Multiple	Facultative	Unknown	37	14	0.37	0.18	1760	86
Streptomyces avermitilis MA-4680	Bacteria	Mesophilic	Multiple	Aerobic	Unknown	25-35 (26)	14	0.71	0.05	7580	176
Streptomyces coelicolor A3(2)	Bacteria	Mesophilic	Multiple	Aerobic	Unknown	25-35	15	0.72	0.04	7769	197
Streptomyces griseus subsp. griseus NBRC 13350	Bacteria	Mesophilic	Multiple	Aerobic	Unknown	25-35	16	0.72	0.05	7136	192
Sulfurimonas denitrificans DSM 1251	Bacteria	Mesophilic	Unknown	Anaerobic	Unknown	20-25	14	0.35	0.08	2096	79
Sulfurovum sp. NBC37-1	Bacteria	Mesophilic	Specialized	Facultative	Unknown	-	12	0.45	0.10	2438	113
Symbiobacterium thermophilum IAM 14863	Bacteria	Thermophilic	Terrestrial	Microaerophilic	NonHalophilic	60	6	0.69	0.03	3338	77

Thermobifida fusca YX	Bacteria	Thermophilic	Multiple	Aerobic	Unknown	50-55	10	0.68	0.06	3110	100
Thermococcus kodakarensis KOD1	Archaea	Hyperthermophilic	Specialized	Anaerobic	Unknown	85	8	0.53	0.11	2306	83
Thermococcus onnurineus NA1	Archaea	Hyperthermophilic	Terrestrial	Anaerobic	Unknown	80	11	0.52	0.10	1976	88
Thermosipho africanus TCF52B	Bacteria	Thermophilic	Specialized	Anaerobic	Unknown	75	7	0.31	0.05	1911	57
Thermosipho melanesiensis BI429	Bacteria	Thermophilic	Unknown	Anaerobic	Unknown	70	5	0.31	0.03	1879	48
Thermus thermophilus HB27	Bacteria	Thermophilic	Specialized	Aerobic	Unknown	68	13	0.7	0.02	1982	59
Thioalkalivibrio sp. HL-EbGR7	Bacteria	Mesophilic	Specialized	Aerobic	ModerateHalophilic	-	5	0.66	0.06	3283	112
Thiobacillus denitrificans ATCC 25259	Bacteria	Mesophilic	Multiple	Facultative	Unknown	28-32	5	0.66	0.04	2827	59
Treponema denticola ATCC 35405	Bacteria	Mesophilic	Host_Associated	Anaerobic	Unknown	30-42	6	0.38	0.06	2767	73
Treponema pallidum subsp. pallidum str. Nichols	Bacteria	Mesophilic	Host_Associated	Anaerobic	Unknown	-	9	0.53	0.03	1036	102
Tropheryma whipplei str. Twist	Bacteria	Mesophilic	Host_Associated	Aerobic	Unknown	37	12	0.46	0.03	808	81
uncultured methanogenic archaeon RC-I	Archaea	Mesophilic	Host_Associated	Unknown	NonHalophilic	-	6	0.56	0.07	3085	81
Ureaplasma parvum serovar 3 str. ATCC 27815	Bacteria	Mesophilic	Host_Associated	Facultative	Unknown	-	15	0.26	0.04	609	49
Ureaplasma parvum serovar 3 str. ATCC 700970	Bacteria	Mesophilic	Host_Associated	Facultative	Unknown	-	15	0.26	0.04	614	50
Ureaplasma urealyticum serovar 10 str. ATCC 33699	Bacteria	Mesophilic	Host_Associated	Facultative	Unknown	37	12	0.26	0.05	646	41
Verminephrobacter eiseniae EF01-2	Bacteria	Mesophilic	Host_Associated	Unknown	NonHalophilic	-	7	0.66	0.03	4908	64
Vibrio cholerae O395	Bacteria	Mesophilic	Multiple	Facultative	Unknown	20-30	16	0.49	0.22	2742	85
Vibrio fischeri MJ11	Bacteria	Mesophilic	Multiple	Facultative	NonHalophilic	20-28	8	0.4	0.16	2590	109
Vibrio harveyi ATCC BAA-1116	Bacteria	Mesophilic	Aquatic	Facultative	ModerateHalophilic	20-30	6	0.46	0.16	3546	135
Vibrio parahaemolyticus RIMD 2210633	Bacteria	Mesophilic	Aquatic	Facultative	Unknown	20-30	7	0.46	0.17	3080	137
Vibrio splendidus LGP32	Bacteria	Mesophilic	Aquatic	Facultative	Unknown	-	6	0.45	0.13	2946	158
Vibrio vulnificus YJ016	Bacteria	Mesophilic	Aquatic	Facultative	Unknown	20-30	12	0.47	0.23	3259	102
Wolbachia endosymbiont of Culex quinquefasciatus Pel	Bacteria	Mesophilic	Host_Associated	Aerobic	Unknown	-	18	0.35	0.04	1275	49
Wolinella succinogenes DSM 1740	Bacteria	Mesophilic	Host_Associated	Microaerophilic	Unknown	-	12	0.49	0.06	2042	75
Xanthobacter autotrophicus Py2	Bacteria	Mesophilic	Multiple	Facultative	Unknown	-	5	0.68	0.03	4746	95
Xanthomonas axonopodis pv. citri str. 306	Bacteria	Mesophilic	Host_Associated	Aerobic	Unknown	25-30	13	0.65	0.07	4312	115
Xanthomonas campestris pv. vesicatoria str. 85-10	Bacteria	Mesophilic	Host_Associated	Aerobic	Unknown	25-30	11	0.65	0.06	4487	118
Xanthomonas oryzae pv. oryzae PXO99A	Bacteria	Mesophilic	Host_Associated	Aerobic	Unknown	-	9	0.64	0.06	4988	141
Xylella fastidiosa 9a5c	Bacteria	Mesophilic	Host_Associated	Aerobic	Unknown	26-28	6	0.54	0.12	2766	163
Yersinia enterocolitica subsp. enterocolitica 8081	Bacteria	Mesophilic	Multiple	Facultative	Unknown	28-30	7	0.48	0.12	3979	102
Yersinia pestis Antiqua	Bacteria	Mesophilic	Multiple	Facultative	NonHalophilic	-	9	0.49	0.12	4167	90
Yersinia pestis biovar Microtus str. 91001	Bacteria	Mesophilic	Multiple	Facultative	Unknown	28-30	9	0.49	0.12	3891	88
Yersinia pseudotuberculosis YPIII	Bacteria	Mesophilic	Multiple	Facultative	Unknown	-	9	0.49	0.12	4192	91

Zymomonas mobilis subsp. mobilis ZM4	Bacteria	Mesophilic	Multiple	Anaerobic	Unknown	25-30	8	0.48	0.11	1998	65

The columns Species, Superkingdom, Temperature Range, Habitat, Oxygen requirement, Salinity, and Optimal temperature give data deduced from the file *ftp.ncbi.nlm.nih.gov/genomes/Bacteria/lproks_0.txt*. HSRG is the number of ribosomal genes occurring in the respective effectome, GC-cont is the GC-content, $\overline{GCB_{Eff}}$ is the mean GCB-value of the effectome, # Genes is the number of analyzed genes and # Genes Effectome is the number of genes belonging to the respective effectome.

Additional file1, Table S2 – Composition of the set Bacteria_HITR

Species	Name	# tRNA Genes	$\overline{GCB_{Eff}}$ -value	d [h]
Photobacterium profundum SS9	NC_006370.gbk	164	0.15	2.5
Shewanella pealeana ATCC 700345	NC_009901.gbk	143	0.14	
Aeromonas hydrophila subsp. *hydrophila*	NC_008570.gbk	128	0.23	0.35
Shewanella woodyi ATCC 51908	NC_010506.gbk	126	0.14	
Vibrio parahaemolyticus RIMD 2210633	NC_004603.gbk	126	0.17	0.2
Shewanella halifaxensis HAW-EB4	NC_010334.gbk	125	0.15	
Shewanella sediminis HAW-EB3	NC_009831.gbk	125	0.17	
Vibrio fischeri MJ11	NC_011184.gbk	124	0.16	0.3
Vibrio splendidus LGP32	NC_011753.gbk	124	0.13	
Vibrio harveyi ATCC BAA-1116	NC_009783.gbk	121	0.16	
Vibrio vulnificus YJ016	NC_005139.gbk	112	0.24	0.16
Aeromonas salmonicida subsp. *salmonicida* A449	NC_009348.gbk	110	0.18	
Bacillus weihenstephanensis KBAB4	NC_010184.gbk	108	0.14	
Alkaliphilus metalliredigens QYMF	NC_009633.gbk	106	0.05	
Pseudoalteromonas haloplanktis TAC125	NC_007481.gbk	106	0.13	0.5
Stenotrophomonas maltophilia K279a	NC_010943.gbk	105	0.08	
Aliivibrio salmonicida LFI1238	NC_011312.gbk	105	0.15	
Bacillus thuringiensis serovar konkukian str. 97-27	NC_005957.gbk	105	0.15	0.42
Shewanella baltica OS195	NC_009997.gbk	104	0.15	
Shewanella sp. ANA-3	NC_008577.gbk	103	0.20	
Shewanella oneidensis MR-1	NC_004347.gbk	102	0.18	0.66
Shigella boydii CDC 3083-94 BS512	NC_010658.gbk	102	0.16	
Shewanella amazonensis SB2B	NC_008700.gbk	101	0.19	
Shewanella putrefaciens CN-32	NC_009438.gbk	101	0.14	
Chromobacterium violaceum ATCC 12472	NC_005085.gbk	98	0.14	0.8
Lactobacillus delbrueckii subsp. *bulgaricus*	NC_008529.gbk	98	0.27	
Symbiobacterium thermophilum IAM 14863	NC_006177.gbk	98	0.03	4.2
Bacillus cereus ATCC 10987	NC_003909.gbk	97	0.17	0.3
Shigella flexneri 2a str. 301	NC_004337.gbk	97	0.16	
Shigella sonnei Ss046	NC_007384.gbk	97	0.16	
Shewanella frigidimarina NCIMB 400 NCBM4000	NC_008345.gbk	96	0.12	
Vibrio cholerae O395	NC_009456.gbk	96	0.22	0.2
Bacillus anthracis str. Ames	NC_003997.gbk	95	0.16	0.5
Shewanella loihica PV-4	NC_009092.gbk	95	0.27	
Clostridium beijerinckii NCIMB 8052	NC_009617.gbk	94	0.10	
Clostridium perfringens ATCC 13124	NC_008261.gbk	93	0.12	0.2
Shewanella denitrificans OS217 OS-217	NC_007954.gbk	93	0.17	

Column 1 gives the name of the species, column 2 the name of the RefSeq dataset. Column 3 lists the number of tRNA genes, column 4 the $\overline{GCB_{Eff}}$ -value and column 5 the minimum generation time deduced from [1] (see below).

Additional file1, Table S3 – Abundance of COG-categories

	COG	Description	Bacteria $\#_{Eff}(Cat)$	Bacteria $\#_{All}(Cat)$	Bacteria $Abund_{Eff}$	Archaea $\#_{Eff}(Cat)$	Archaea $\#_{All}(Cat)$	Archaea $Abund_{Eff}$
Information storage & processing	J	Translation, ribosomal structure and biogenesis	15176	33907	0.79	523	1850	0.56
	K	Transcription	1159	64085	-0.60	18	1184	-0.71
	L	Replication, recombination and repair	188	25504	-0.99	9	590	-0.71
Metabolism	C	Energy production and conversion	24052	265676	0.10	865	9251	0.08
	E	Amino acid transport and metabolism	996	41872	-0.48	49	1565	-0.40
	F	Nucleotide transport and metabolism	1424	23008	-0.07	52	1006	-0.18
	G	Carbohydrate transport and metabolism	2050	51374	-0.26	22	871	-0.44
	H	Coenzyme transport and metabolism	184	23638	-0.97	18	1004	-0.64
	I	Lipid transport and metabolism	515	20562	-0.46	6	371	-0.68
	P	Inorganic ion transport and metabolism	161	10120	-0.66	6	384	-0.70
	Q	Secondary metabolites biosynthesis, transport and catabolism	5	719	-1.01	0	22	-
Cellular processes	D	Cell cycle control, cell division, chromosome partitioning	8	1729	-1.12	0	4	-
	M	Cell wall/membrane/envelope biogenesis	25516	352866	0.00	952	12409	-0.01
	N	Cell motility	540	15546	-0.32	8	228	-0.35
	O	Posttranslational modification, protein turnover, chaperones	386	36940	-0.84	17	1109	-0.71
	T	Signal transduction mechanisms	513	40556	-0.76	2	670	-1.42
		Sum:	72873	1008102		2547	32518	

In analogy to the mapping of FunCat categories, we mapped GO-Terms onto COG-Categories [2] (see below). For archaeal and bacterial genomes, the number of gene products contributing to COG categories was determined. The column labeled "$\#_{All}(Cat)$" gives the number of genes deduced from the whole dataset. The column labeled "$\#_{Eff}(Cat)$" gives the number of genes belonging to the respective effectomes. The column "$Abund_{Eff}$" lists the ratio $\log(f_{Eff}(Cat)/f_{All}(Cat))$ for the category, if $\#_{All}(Cat)$ was at least 100. In all other cases a trend is given indicated by a "-" for underrepresentation. The line labeled "Sum" lists the number of genes being analyzed.

Additional file1, Table S4 - Content of the genomic datasets used

Content	Name of Dataset	# Genomes	# Genes
All Archaea	*MG_CUB*(Archaea)	18	39092
Hyperthermophilic Archaea	*MG_CUB*(Archaea_HT)	7	13332
Mesophilic Archaea	*MG_CUB*(Archaea_MS)	10	24158
Aquatic Archaea	*MG_CUB*(Archaea_AQU)	9	18168
Anaerobic Archaea	*MG_CUB*(Archaea _ANE)	14	28370
All Bacteria	*MG_CUB*(Bacteria)	370	1175058
Thermophilic Bacteria	*MG_CUB*(Bacteria_TH)	15	35901
Mesophilic Bacteria	*MG_CUB*(Bacteria_MS)	315	1023229
Psychrophilic Bacteria	*MG_CUB*(Bacteria_PS)	13	37973
Aquatic Bacteria	*MG_CUB*(Bacteria _AQU)	44	158454
Terrestrial Bacteria	*MG_CUB*(Bacteria_TER)	28	115958
Aerobic Bacteria	*MG_CUB*(Bacteria_AER)	139	518452
Anaerobic Bacteria	*MG_CUB*(Bacteria_ANE)	47	12778
Host-associated Bacteria	*MG_CUB*(Bacteria_HOA)	129	319508
Non-halophilic Bacteria	*MG_CUB*(Bacteria_NHAL)	78	252700
Moderate halophilic Bacteria	*MG_CUB*(Bacteria_MHAL)	8	27332
Extremely optimized Bacteria	*MG_CUB*(Bacteria_HITR)	37	145830

Column 1 describes the content of the datasets. Their names are listed in column 2. Columns 3 and 4 give the number of genomes and genes being contained in the datasets.

Literature cited

1. Vieira-Silva S, Rocha EP: **The systemic imprint of growth and its uses in ecological (meta)genomics**. *PLoS Genet* 2010, **6**(1):e1000808.
2. Tatusov RL, Fedorova ND, Jackson JD, Jacobs AR, Kiryutin B, Koonin EV, Krylov DM, Mazumder R, Mekhedov SL, Nikolskaya AN *et al*: **The COG database: an updated version includes Eukaryotes**. *BMC Bioinformatics* 2003, **4**(1):41.
