# Supplementary material for: Genes optimized by evolution for accurate and fast translation encode in Archaea and Bacteria a broad and characteristic spectrum of protein functions
Source: BMC Genomics. 2010 Nov 4;11:617. doi: 10.1186/1471-2164-11-617 (PMC3091758; doi:10.1186/1471-2164-11-617)
Supplement: Additional file 5 — Figure S1 - Glycolysis/Gluconeogenesis as depicted in the reference pathway of KEGG. [file 1471-2164-11-617-S5.PDF]

## Glycolysis / Gluconeogenesis - Reference pathway (KO)

[ Pathway menu | Pathway entry | Show description ]

Reference pathway (KO) Go

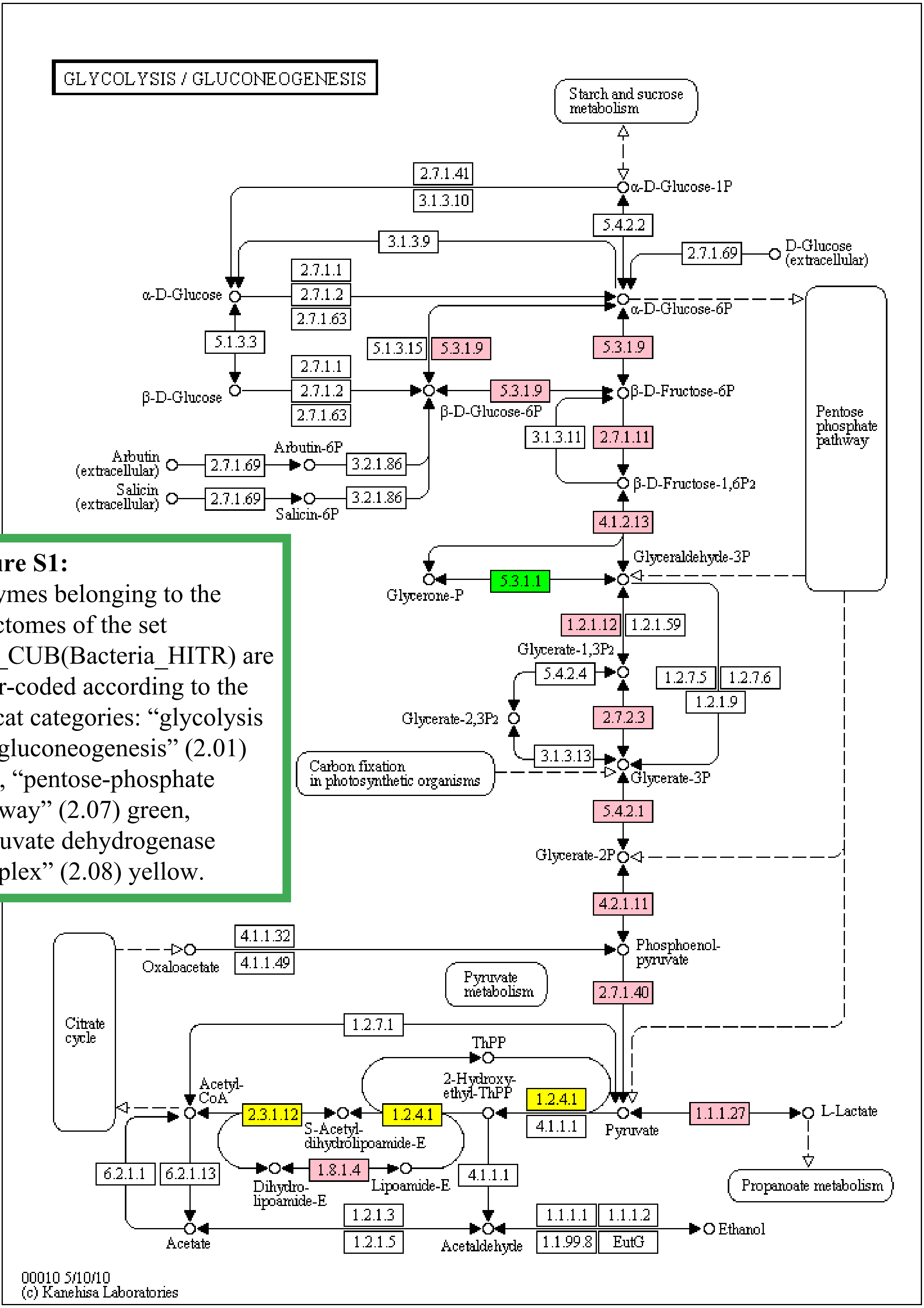


**Figure S1:** Enzymes belonging to the effectomes of the set MG_CUB(Bacteria_HITR) are color-coded according to the Funcat categories: "glycolysis and gluconeogenesis" (2.01) pink, "pentose-phosphate pathway" (2.07) green, "pyruvate dehydrogenase complex" (2.08) yellow.
