# Supplementary material for: Genes optimized by evolution for accurate and fast translation encode in Archaea and Bacteria a broad and characteristic spectrum of protein functions
Source: BMC Genomics. 2010 Nov 4;11:617. doi: 10.1186/1471-2164-11-617 (PMC3091758; doi:10.1186/1471-2164-11-617)
Supplement: Additional file 6 — Figure S2 - TCA cycle as depicted in the reference pathway of KEGG. [file 1471-2164-11-617-S6.PDF]

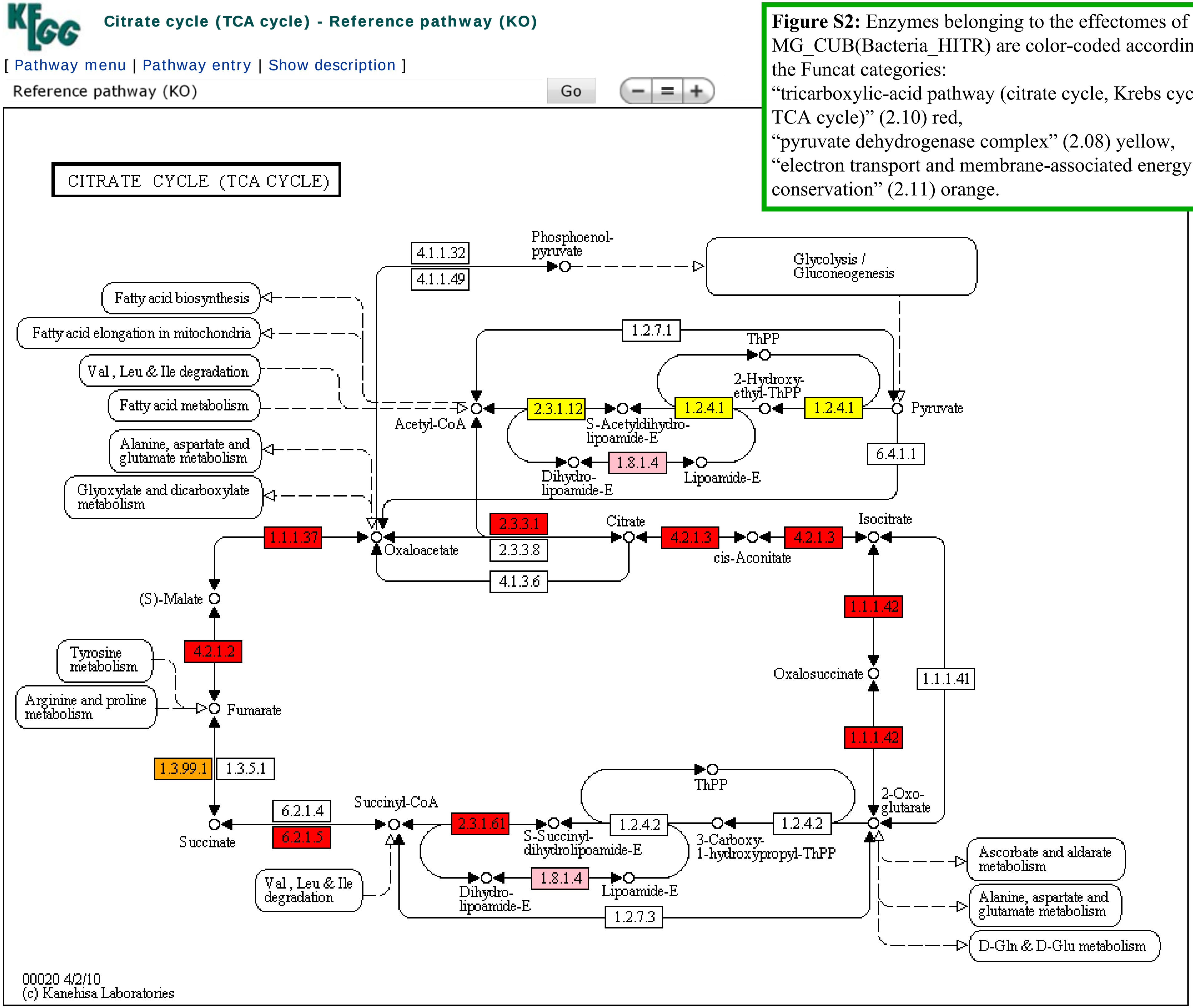

Figure S2: Enzymes belonging to the effectomes of the set MG_CUB(Bacteria_HITR) are color-coded according to the Funcat categories:
“tricarboxylic-acid pathway (citrate cycle, Krebs cycle, TCA cycle)” (2.10) red,
“pyruvate dehydrogenase complex” (2.08) yellow,
“electron transport and membrane-associated energy conservation” (2.11) orange.
